# Synthesis, Structure and Cytotoxic Properties of Copper(II) Complexes of 2-Iminocoumarins Bearing a 1,3,5-Triazine or Benzoxazole/Benzothiazole Moiety

**DOI:** 10.3390/molecules27217155

**Published:** 2022-10-22

**Authors:** Anna Makowska, Franciszek Sączewski, Patrick J. Bednarski, Maria Gdaniec, Łukasz Balewski, Magdalena Warmbier, Anita Kornicka

**Affiliations:** 1 Department of Chemical Technology of Drugs, Faculty of Pharmacy, Medical University of Gdańsk, Al. Gen. J. Hallera 107, 80-416 Gdansk, Poland; 2 Department of Pharmaceutical and Medicinal Chemistry, Institute of Pharmacy, University of Greifswald, F.-L. Jahn Strasse 17, D-17489 Greifswald, Germany; 3 Faculty of Chemistry, Adam Mickiewicz University, ul. Uniwersytetu Poznańskiego 8, 61-614 Poznań, Poland

**Keywords:** 2-iminocoumarins, 2-imino-2*H*-chromene, 2*H*-chromen-2-imines, 1,3,5-triazines, benzoxazoles, benzothiazoles, copper(II) complexes, X-ray analysis, in vitro cytotoxic activity

## Abstract

A series of copper(II) complexes of 2-imino-2*H*-chromen-3-yl-1,3,5-triazines **2a-h**, 3-(benzoxazol-2-yl)-2*H*-chromen-2-imines **4a-b**, and 3-(benzothiazol-2-yl)-2*H*-chromen-2-imines **6a-c** were obtained by reacting of appropriate 2-iminocoumarin ligands **L1a-h**, **L3a-b**, and **L5a-c** with 3-fold molar excess of copper(II) chloride. The structure of these compounds was confirmed by IR spectroscopy, elemental analysis, and single-crystal X-ray diffraction data (**2f**, **2g**, **2h**, and **6c**). All the synthesized complexes were screened for their activity against five human cancer cell lines: DAN-G, A-427, LCLC-103H, SISO, and RT-4 by using a crystal violet microtiter plate assay and relationships between structure and in vitro cytotoxic activity are discussed. The coordination of 2-iminocoumarins with copper(II) ions resulted in complexes **2a-h**, **4a-b**, and **6a-c** with significant inhibitory properties toward tested tumor cell lines with IC_50_ values ranging from 0.04 μM to 15.66 μM. In comparison to the free ligands **L1a-h**, **L3a-b**, and **L5a-c**, the newly prepared Cu(II) complexes often displayed increased activity. In the series of copper(II) complexes of 2-imino-2*H*-chromen-3-yl-1,3,5-triazines **2a-h** the most potent compound **2g** contained a 4-phenylpiperazine moiety at position 6 of the 1,3,5-triazine ring and an electron-donating diethylamino group at position 7′ of the 2-iminocoumarin scaffold. Among the Cu(II) complexes of 3-(benzoxazol-2-yl)-2*H*-chromen-2-imines **4a-b** and 3-(benzothiazol-2-yl)-2*H*-chromen-2-imines **6a-c** the most active was benzoxazole-2-iminocoumarin **4b** that also possessed a diethylamino group at position 7′ of the 2-iminocoumarin moiety. Moreover, compound **4b** was found to be the most prominent agent and displayed the higher potency than cisplatin against tested cell lines.

## 1. Introduction

Cancer—a multifactorial disease—constitutes the second leading cause of worldwide mortality, being a major public health issue. Despite the fact that there are several various approaches in cancer treatment, chemotherapy still remains one of the most important strategies [1]. Among clinically approved antineoplastic drugs cisplatin and its analogues have a leading and well-established position in the treatment of cancer. Unfortunately, their use is limited by toxicity and severe side effects [2]. In addition to high toxicity, another serious problem is acquired resistance of cancer cells to platinum drugs [3,4]. Therefore, there is a need for novel transition metal-complexes as a drug-candidates with less undesirable effects.

Among transition metals, copper occupies an important position with respect to its presence in living organisms and biological role in physiological processes, such as cell metabolism or mitochondrial respiration [5,6,7,8]. Moreover, copper displays a redox-active properties and possess an ability to form stable complexes with diverse ligands containing donor atoms, such as nitrogen, sulfur, or oxygen. In the field of medicinal chemistry, it has been confirmed that metal complexes may possess a higher biological activity, selectivity, and lower toxicity compared to the free ligands [9,10]. It was found that the uptake of copper complexes by normal cells is lower than by tumor cells [11]. Therefore, considerable research has been devoted to the synthesis of copper(II) complexes with anticancer properties [12,13,14,15,16,17].

The antiproliferative effects of the copper(II) complexes may result from various mechanisms. For example, copper(II) complexes with a well-defined antitumor mechanism of action include intercalators of DNA [18,19,20,21,22,23], inhibitors of protein disulfide isomerase (PDI) [24] or topoisomerases I and II (dual Top1/Top2α inhibitors) [25,26], as well as inductors of p53-dependent apoptosis [27]. Copper compounds with SOD-mimicking properties are of particular interest since oxidative stress is involved in cancers or neurodegenerative disorders which result from an imbalance of ROS concentration [28]. The antiproliferative effects of copper complexes may also result from their ability to induce ROS generation that trigger tumor cell death [29] or antiangiogenic properties [30]. Additionally, copper complexes were extensively explored due to their antimicrobial [31,32], antifungal [33], antiviral activity [34], and anti-inflammatory properties [35,36,37,38].

Several compounds with a central 2-iminocoumarin (2-imino-2*H*-chromene)—less known coumarin analogs—are studied for many interesting potential applications due to their optical or fluorescent properties [39,40,41,42,43,44,45,46,47,48,49,50,51,52]. Furthermore, it was found that compounds bearing 2-iminocoumarin core possess antimicrobial [53,54,55] and anti-inflammatory [56] activities, or neuroprotective properties, such as BACE1 inhibitors [57]. Worth noting is the fact that 2-iminocoumarins have been reported as potential anticancer agents [58,59,60,61,62], whereas their metal complexes have been described as antioxidants and antimicrobials [63,64]. Nevertheless, the research of biological activity of 2-iminocoumarins, especially their metal complexes, is not very extensive.

**Figure 1:**
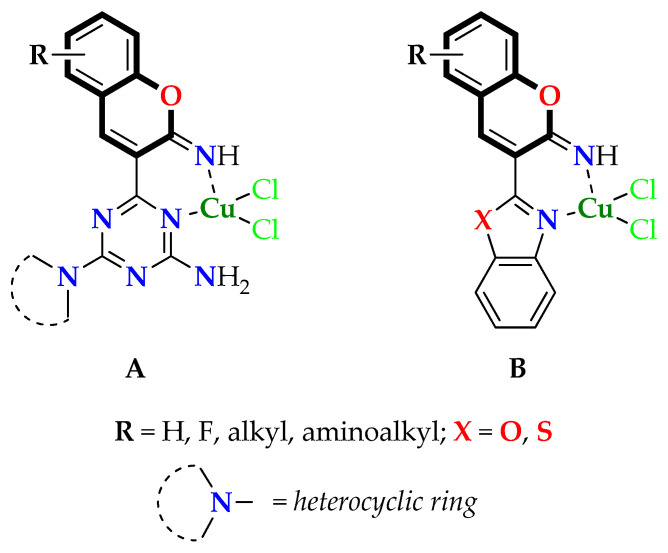
General structure of designed copper(II) complexes of 2-iminocoumarins bearing 1,3,5-triazine ring **A** and benzoxazole or benzothiazole moiety **B**.

Our previous studies on the synthesis and cytotoxic evaluation of 2-iminocoumarins bearing 2,4-diamino-1,3,5-triazine [65], benzoxazole, or benzothiazole moiety [66] showed that such hybrid molecules exhibit pronounced anticancer activity against several human tumor cell lines. Continuing the search for compounds with an expectation of higher antitumor activity, we focused on the synthesis of copper(II) complexes of the above-mentioned hybrid molecules composed of 2-iminocoumarins and 2,4-diamino-1,3,5-triazines **A** or benzoxazole/benzothiazole-2-iminocoumarins **B** (Figure 1).

## 2. Results and Discussion

### 2.1. Chemistry

#### 2.1.1. Synthesis of Copper(II) Complexes of 2-imino-2*H*-chromen-3-yl-1,3,5-triazine Derivatives **2a-h**

**Scheme 1:**
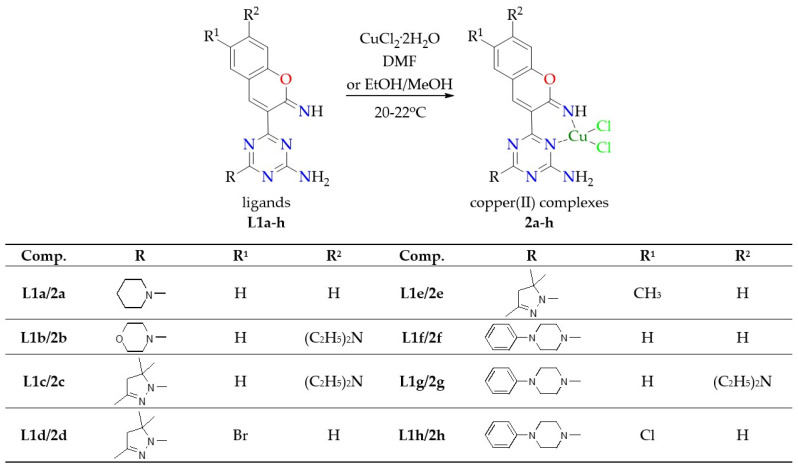
Synthesis of copper(II) complexes of 2-imino-2*H*-chromen-3-yl-1,3,5-triazines **2a**-**h**.

Copper(II) complexes of 2-imino-2*H*-chromen-3-yl-1,3,5-triazine derivatives **2a-h** were prepared by reaction of copper(II) chloride dihydrate with previously described ligands **L1a**-**h** [65]. The reactions were carried out at 20–22 °C in solvents such as dimethylformamide, ethanol, or methanol, containing 1–2% of water (Scheme 1). Dimethylformamide turned out to be the most convenient due to better solubility of 2-iminocoumarin ligands.

Dark brown, orange, violet, or green crystals of copper(II) complexes were obtained upon slow evaporation of the solvent over 1–4 days. It should be noted that this process in some cases resulted in the isolation of a significant amount of coumarin as a result of the hydrolysis of the imine moiety. For this reason, dimethylformamide was replaced with lower boiling ethanol (compounds: **2b**, **2c**, and **2f**) or methanol (compounds: **2e**, **2g**, and **2h**).

The best yields (26–27%) were achieved with ligands **L1b** and **L1g** containing morpholine or phenylpiperazine rings (R = morpholine, phenylpiperazine) and an electron-donating substituent—diethylamino group (R^1^ = H, R^2^ = (C_2_H_5_)_2_N). It was found that the best results were obtained using appropriate ligand and copper(II) chloride in a molar ratio of 1:3. Stoichiometric amounts, as well as 1:2 ratio of ligand, copper(II), and copper salt, led to the products constituting a mixture of the 2-iminocoumarin and coumarin complexes.

**Figure 2:**
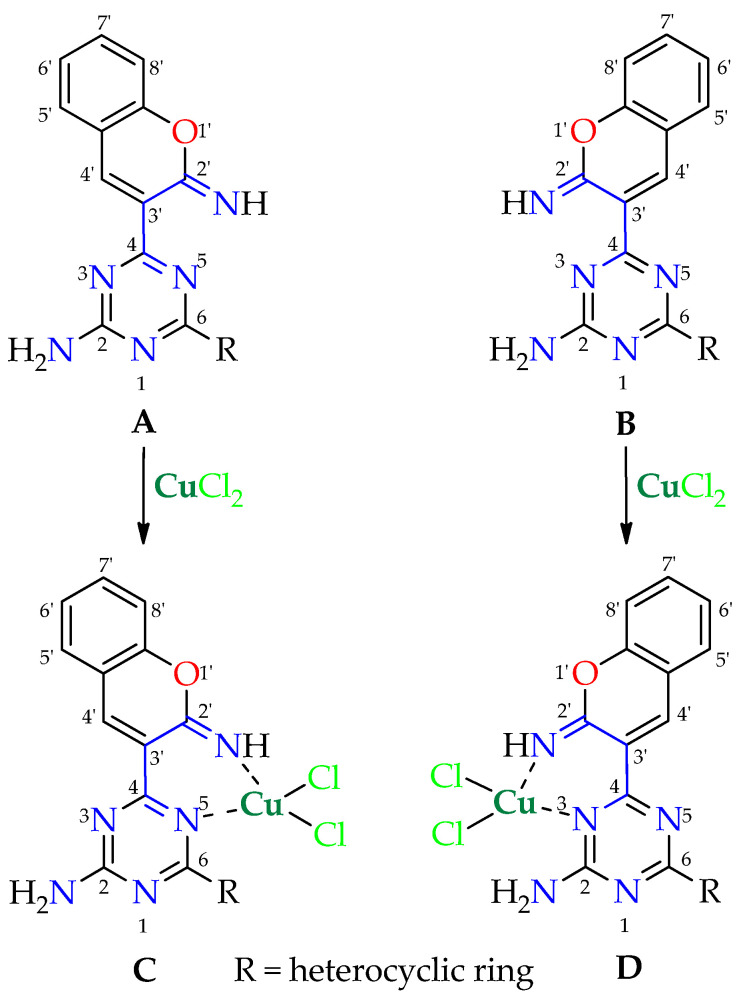
Two possible conformations **C** and **D** of copper(II) complexes of 2-iminocoumarin-1,3,5-triazines.

**Figure 3:**
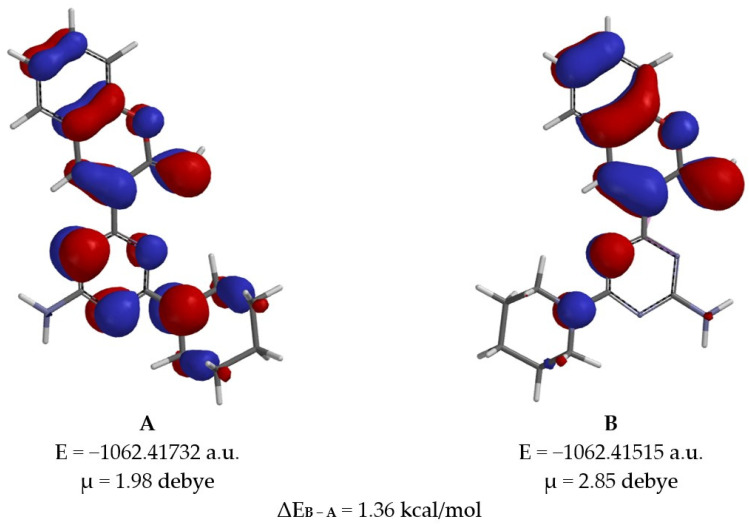
Conformational structures **A** and **B** of ligand **L1a** optimized by density functional method (B3LYP/6-31G*) using Spartan version ‘14 V 1.1.4’. The location of the HOMO orbital is marked in red and blue.

Copper(II) complexes of 2-iminocoumarin are formed by self-assembly of a mixture of copper(II), chloride, and appropriate ligand containing nitrogen donor atoms of 2-iminocoumarin skeleton and the 1,3,5-triazine ring. Formally, we are dealing here with a β-diimine system (β-*NN*) and due to the conjugation of π electrons of both heterocyclic rings, we may consider two possible conformations of copper(II) complex (structures **C** and **D**, as shown in Figure 2. Thus, the reaction of 2-iminocoumarins **A** and **B** may lead to complexes in which the central copper atom is chelated by a bidentate neutral ligand forming a six-membered ring -Cu-N_5_-C_4_-C_3′_-C_2′_-N (structure **C**) or -Cu-N_3_-C_6_-C_3′_-C_2′_-N (structure **D**). Therefore, to obtain better insight into the structure of the ligands used for complexation with copper salt, the relative stability of conformers **A** and **B** for ligand **L1a** (Figure 2) containing piperidine moiety was determined using the Spartan computer program (version ‘14 V 1.1.4.’). The obtained data revealed that conformer **A** was calculated to be slightly lower in energy than conformer **B** (ΔE = 1.36 kcal/mol). However, based on their calculated dipole moments, the conformer **B** (μ = 2.85 debye) would be predicted to predominate over the conformer **A** (μ = 1.98 debye) in a polar solvent used for the reaction—ethanol or dimethylformamide (Figure 3). It should be noted, that more stable conformer **A** upon complexation gives rise the formation of mononuclear copper(II) complex (structure **C**, Figure 2), where central metal atom is chelated by neutral 2-iminocoumarin-1,3,5-triazine ligand with formation of six-membered ring involving two nitrogen atoms—at position 5 of heterocyclic ring and imine moiety (C_2_=NH), respectively.

#### 2.1.2. Synthesis of Copper(II) Complexes of 3-(benzoxazol-2-yl)-2*H*-chromen-2-imines **4a-b** and 3-(benzothiazol-2-yl)-2*H*-chromen-2-imines **6a-c**

**Scheme 2:**
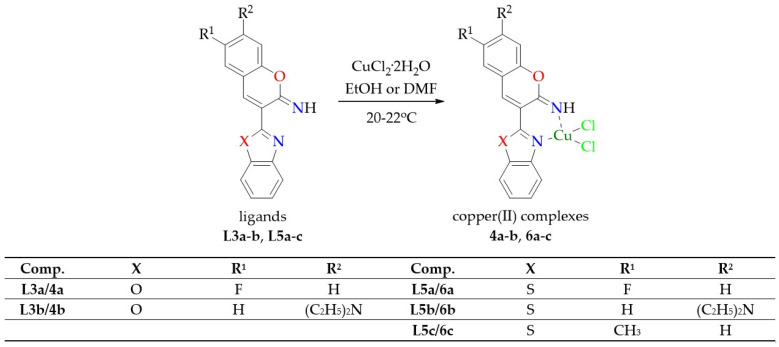
Synthesis of copper(II) complexes of 3-(benzoxazol-2-yl)-2*H*-chromen-2-imines **4a**-**b** and 3-(benzothiazol-2-yl)-2*H*-chromen-2-imines **6a**-**c**.

Copper(II) complexes of 3-(benzoxazol-2-yl)-2*H*-chromen-2-imines **4a**-**b** and 3-(benzothiazol-2-yl)-2*H*-chromen-2-imines **6a**-**c** were obtained by the reaction of appropriate ligands **L3a-b** or **L5a-c** [66] with copper(II) chloride dihydrate in 98% ethanol or dimethylformamide. Violet-dark or green crystals were collected after slow evaporation of the solvent at room temperature (20–22 °C) over 24 h to 4 days (Scheme 2).

### 2.2. Structure of Copper(II) Complexes of 2-imino-2H-chromen-3-yl-1,3,5-triazines ***2a-h***, 3-(benzoxazol-2-yl)-2H-chromen-2-imines ***4a-b***, and 3-(benzothiazol-2-yl)-2H-chromen-2-imines ***6a-c***

**Figure 4:**
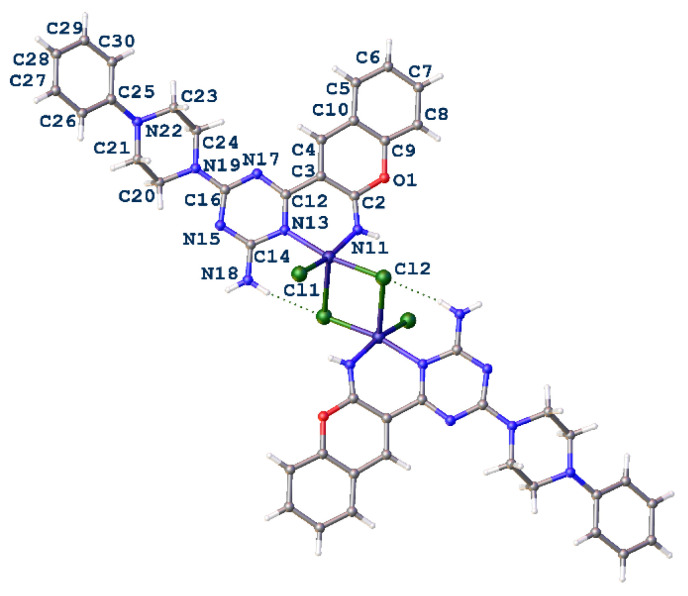
The dimeric molecular structure of **2f**. Displacement ellipsoids are shown at the 50% probability level.

**Figure 5:**
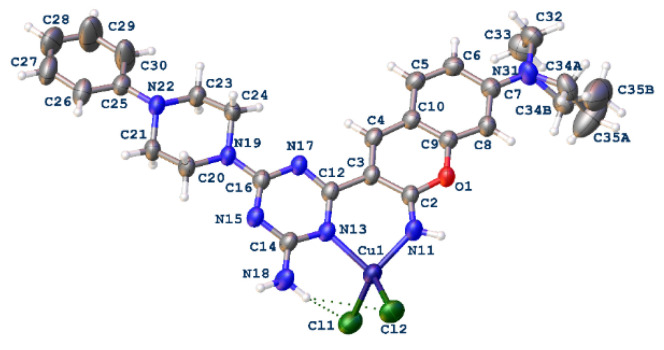
Molecular structure of **2g**. Displacement ellipsoids are shown at the 50% probability level.

**Figure 6:**
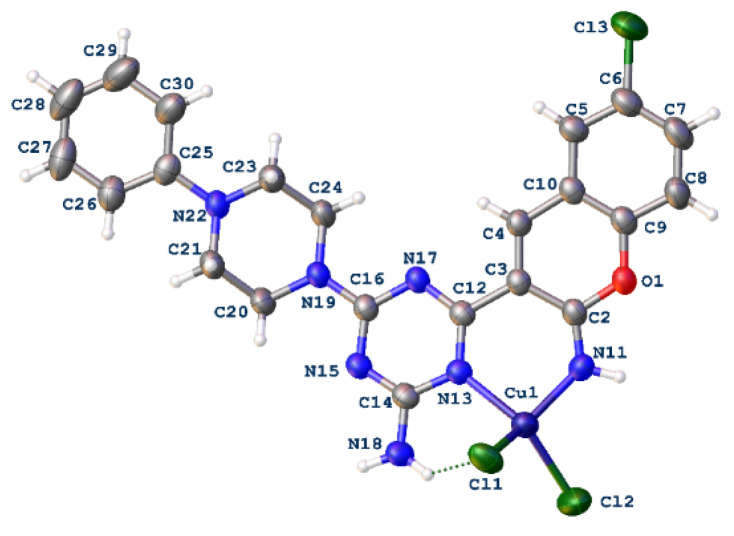
Molecular structure of **2h**. Displacement ellipsoids are shown at the 50% probability level.

**Figure 7:**
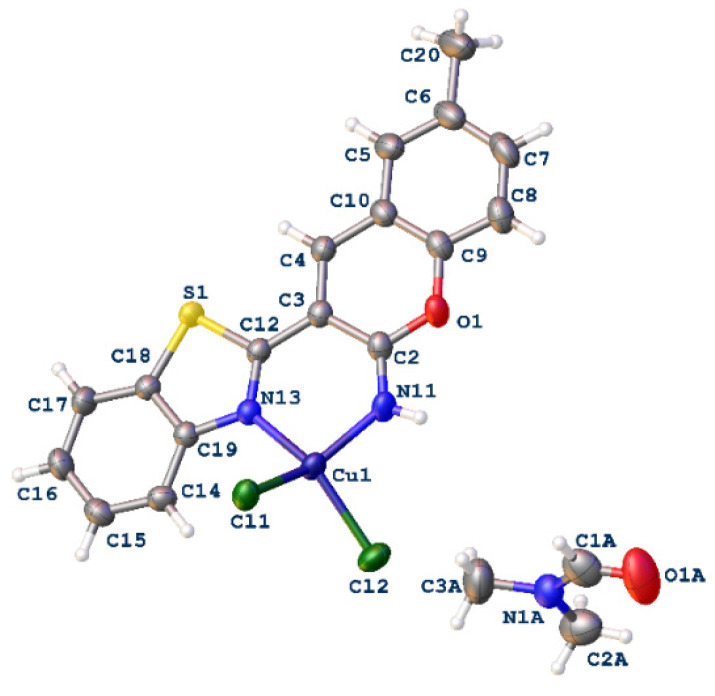
The asymmetric unit of **6c**. Displacement ellipsoids are shown at the 50% probability level.

The structures of the copper(II) complexes of 2-imino-2*H*-chromen-3-yl-1,3,5-triazines **2a**-**h** or 3-(benzoxazol-2-yl)-2*H*-chromen-2-imines **4a**-**b** and 3-(benzothiazol-2-yl)-2*H*-chromen-2-imines **6a**-**c** were confirmed by elemental analysis, infrared spectroscopic data, as well as X-ray crystallographic studies for **2f**, **2g**, **2h**, and **6c** (Figure 4, Figure 5, Figure 6 and Figure 7).

In the IR spectra of copper(II) complexes of 2-imino-2*H*-chromen-3-yl-1,3,5-triazine derivatives **2a-h** a strong band in a rage of 3110 to 3488 cm^−1^ is assigned to the N-H stretching vibrations of the −C=N-H group and the primary amine group (NH_2_) of the 1,3,5-triazine ring. On the other hand, a broad absorption observed at 3209–3392 cm^−1^ in the IR spectra of the free ligands **L1a-h** [65], has disappeared in the IR spectra of complexes **2a-h**. In the discussed spectra of complexes **2a-h** a strong absorption attributable to the −C=N- of the −C=N-H group occurs in the range of 1643–1654 cm^−1^ and is shifted toward higher or lower wavenumbers in comparison with the spectra of the free ligands **L1a-h** (1649–1672 cm^−1^) [65]. In turn, spectroscopic characterization of the copper(II) complexes of 3-(benzoxazol-2-yl)-2*H*-chromen-2-imines **4a-b** and 3-(benzothiazol-2-yl)-2*H*-chromen-2-imines **6a-c** shows that the N-H of the −C=N-H group vibrational stretching modes appear in the 3119 to 3292 cm^−1^ and 3178 to 3338 cm^−1^ regions, and are shifted toward lower or higher wavenumbers in comparison with the IR spectra of the free ligands **L3a-b** and **L5a-c** (3290–3279 cm^−1^ and 3213–3230 cm^−1^, respectively) [66]. The vibration modes of ν(C=N) of the −C=N-H group appear in the ranges of 1635–1654 cm^−1^ and 1636–1664 cm^−1^. In general, these frequencies shift toward lower frequencies in comparison with those of the free ligands **L3a-b** and **L5a-c** (1655–1663 cm^−1^ and 1662–1664 cm^−1^, respectively) [66].

It should be noted that the presence of copper(II) ions, that can act as Lewis acid in solution, could facilitate the hydrolysis of the 2-iminocoumarin ligands under experimental conditions. On the other hand, due to the presence of an unpaired electron resulting in paramagnetic properties of copper(II) complexes, the nuclear magnetic resonance (NMR) spectra cannot be registered. Thus, the great emphasis was placed on obtaining the single crystals suitable for X-ray diffraction studies to confirm structures of synthesized copper(II) complex compounds.

In all studied compounds, the organic ligands **L** act as bidentate *N*,*N*-chelates forming neutral mononuclear [**L**CuCl_2_] or binuclear [(**L**CuCl)_2_(μ-Cl)_2_] complexes with CuCl_2_. In complexes **2f**, **2g**, and **2h**, the ligand on binding to the metal ion adopts the B conformation. In mononuclear complexes **2h**, **2g,** and **6c,** the Cu (II) atom is tetracoordinated with the coordination environment intermediate between tetrahedral and square planar as indicated by the analysis with the SHAPE program (version 2.1). In the dinuclear complex, **2f,** the pentacoordinated metal center is in a slightly distorted tetragonal pyramidal environment. In this compound, one of the Cl ligands is acting in a bridging mode binding asymmetrically the metal centers in the Cu_2_Cl_2_ coordination ring [Cu-Cl distances are 2.3330(6) and 2.6959(7) Å]. The bond formed by the metal center with the primary imine group N atom of the 2-iminocoumarin fragment, which is in the range 1.930(2)–1.957(2) Å, is always shorter than the bond to the secondary imine group N atom in the chelate ring (1.994(2)–2.053(2) Å).

In **2f**, **2h,** and **2g** primary NH_2_ amino groups are involved in intramolecular N-H···Cl hydrogen bonds and intermolecular N-H···N or N-H···Cl interactions, whereas the imino =N-H group does not take part in any hydrogen bonding. In the case of **6c**, where the primary NH_2_ group is missing, the imino C=N-H proton takes part in N-H···Cl interaction assembling the coordination molecules into hydrogen-bonded centrosymmetric dimer, where the distance between the two-metal center is 3.9916(5) Å. 

Some additional geometrical details can be found in the Supplementary Materials.

### 2.3. UV-Vis Studies of Copper(II) Complexes of 2-imino-2H-chromen-3-yl-1,3,5-triazines ***2a-h***, 3-(benzoxazol-2-yl)-2H-chromen-2-imines ***4a-b***, and 3-(benzothiazol-2-yl)-2H-chromen-2-imines ***6a-c*** in Aqueous Buffer Solution

**Figure 8:**
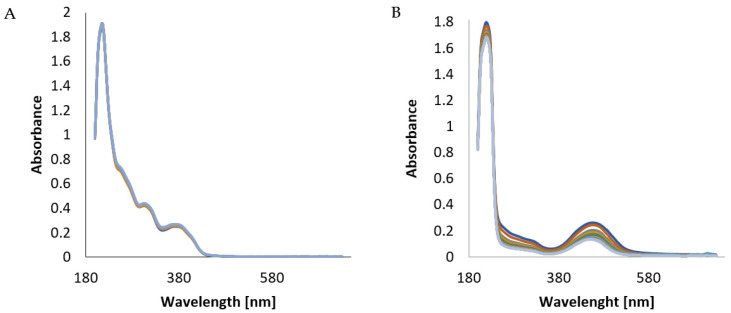
UV-Vis spectra of compounds: (**A**) **2d** and (**B**) **2b** in 0.1% DMF/PBS solution (pH 7.4) at a concentration of 40 μM and 37 °C.

The chemical stability of copper(II) complexes **2a**-**h**, **4a**-**b**, and **6a**-**c** in phosphate-buffered saline solution (PBS, Dulbecco’s buffer, pH 7.4) at 37 °C was investigated using UV-Vis spectroscopy. The Figure 8 shows the time-dependent changes in the UV-Vis spectra of the selected complexes ***2*b** and ***2*d** over 6 h when they were incubated at 37 °C in PBS. In general, all copper(II) complexes tested proved to be relatively stable in the PBS solution, as exemplified by the complexes **2b** and **2d**, since no new spectra with the formation of isosbestic points were observed. The complex **2d** (Figure 8A) showed no noticeable time-dependent changes, whereas complex ***2*b** (Figure 8B) displayed a decrease in the intensity of the initial spectrum, but no change in the shape of the spectrum.

### 2.4. In Vitro Cytotoxic Activity of Copper(II) Complexes of 2-imino-2H-chromen-3-yl-1,3,5-triazines ***2a-h***, 3-(benzoxazol-2-yl)-2H-chromen-2-imines ***4a-b***, and 3-(benzothiazol-2-yl)-2H-chromen-2-imines ***6a-c***

All copper(II) complexes of 2-imino-2*H*-chromen-3-yl-1,3,5-triazines **2a**-**h** and 3-(benzoxazol-2-yl)-2*H*-chromen-2-imines **4a**-**b** and 3-(benzothiazol-2-yl)-2*H*-chromen-2-imines **6a**-**c** were investigated for antiproliferative activity in a panel of five human cancer cell lines of different origin including: human pancreas adenocarcinoma DAN-G, human lung carcinoma A-427, human non-small cell lung cancer LCLC-103H, human cervix cancer SISO, and human urinary bladder carcinoma RT-4.

**Figure 9:**
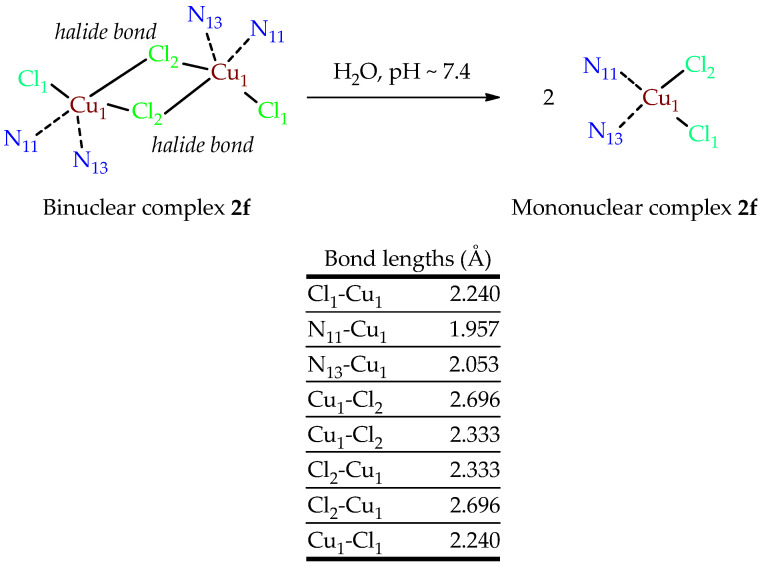
Selected bond lengths in the complex **2f**.

In regards to the cytotoxicity studies, it should be mentioned that the bond length of the halide bridge (Cu_1_-Cl_2_) of copper(II) complex **2f** is 2.696 Å (Figure 9). Compared to the Cu-Cl bond lengths observed in the mononuclear complexes **2g-h** (2.24–2.33 Å), the dimer **2f** under physiological conditions can easily dissociate to form a mononuclear copper(II) complex with the structure analogous to the discussed complexes **2a**-**e** and **2g**-**h**. Thus, it can be assumed that in the solution, the tested complex **2f** possesses a similar structure to that of the complexes **2a-e** and **2g-h**.

**Figure 10:**
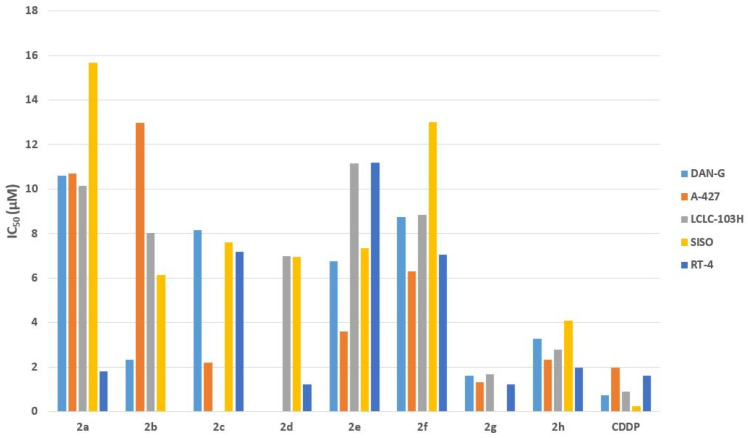
IC_50_ (µM) values of copper(II) complexes of 2-imino-2*H*-chromen-3-yl-1,3,5-triazines **2a**-**h** on five human tumor cell lines (DAN-G, A-427, LCLC-103H, SISO, RT-4) compared to cisplatin (CDDP).

**Figure 11:**
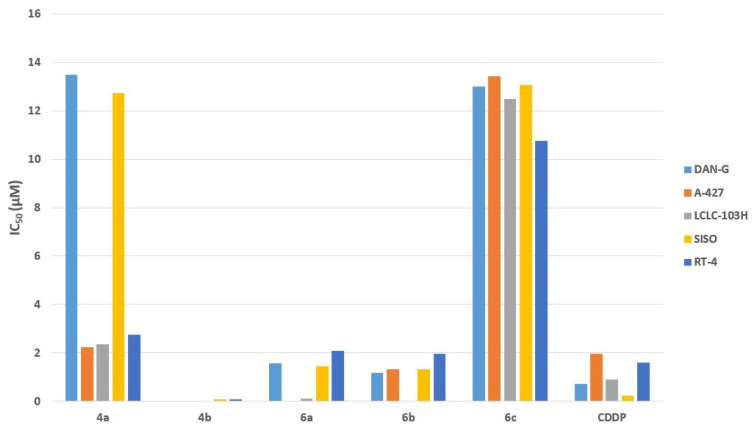
IC_50_ (µM) values of copper(II) complexes of 3-(benzoxazol-2-yl)-2*H*-chromen-2-imines **4a**-**b** and 3-(benzothiazol-2-yl)-2*H*-chromen-2-imines **6a**-**c** on five human tumor cell lines (DAN-G, A-427, LCLC-103H, SISO, RT-4) compared to cisplatin (CDDP).

The IC_50_ values of the tested copper(II) complexes, **2a**-**h**, **4a**-**b**, and **6a**-**c,** together with the data obtained for previously reported free ligands, 2-imino-2*H*-chromen-3-yl-1,3,5-triazines **L1a**-**h** [65], 3-(benzoxazol-2-yl)-2*H*-chromen-2-imines **3a**-**b**, and 3-(benzothiazol-2-yl)-2*H*-chromen-2-imines **5a**-**c** [66], are presented in Table 1 and Table 2 and Figure 10 and Figure 11, respectively.

As revealed by the data in Table 1, the copper(II) complexes of 2-imino-2*H*-chromen-3-yl-1,3,5-triazines **2a**-**h** displayed similar or even higher growth inhibitory potency than that of their free ligands **L1a**-**h** (IC_50_ = 1.21–15.66 µM vs. 1.51–37.19 µM). The highest difference in antiproliferative activity was shown by 4-phenylpiperazine-containing complex **2h** (R = 4-phenylpiperazine) with a chlorine atom at position 6′ (R^1^ = Cl) of the 2-iminocoumarin moiety (compare **2h** with **L1h**: IC_50_ = 1.97–4.09 µM vs. 23.26–37.19 µM).

Similarly to the free ligands **L1a**-**h** [65], among the tested compounds, the most potent complex was the compound **2g** bearing a bulky lipophilic 4-phenylpiperazine moiety at position 6 of the 1,3,5-triazine ring (R = 4-phenylpiperazine) and an electron-donating diethylamino group at position 7′ of the 2-iminocoumarin skeleton (R^2^ = (C_2_H_5_)_2_N), which showed slightly lower cytotoxic activity than the reference cisplatin in the DAN-G, A-427, LCLC-103H and RT-4 cell lines (IC_50_ = 1.21–1.66 µM vs. 0.73–1.96 µM) (Table 1, Figure 10). On the other hand, the least active in this series was analogue **2a** containing piperidine moiety at position 6 of the 1,3,5-triazine ring (R = piperidine), and the unsubstituted 2-iminocoumarin scaffold (R^1^ = R^2^ = H) (IC_50_ = 1.82–15.66 µM) (Table 1, Figure 10).

In previous work we showed that a significant antiproliferative activity of 2-iminocoumarin-1,3,5-triazines is associated with the diethylamino substituent at position 7′ of the 2-iminocoumarin scaffold (R^2^ = (C_2_H_5_)_2_N) [65]. However, from the data presented in the Table 1 and Figure 10, it is apparent that not all active Cu (II) complexes of 2-iminocoumarin-1,3,5-triazines possess an electron-donating diethylamino group (R = (C_2_H_5_)_2_N) at position 7′ of the 2-iminocoumarin moiety. For example, within the series of Cu(II) complexes **2c**-**e** containing a pyrazoline moiety (R = pyrazoline) at position 6 of the 1,3,5-triazine ring, derivatives **2c** and **2d** with an electron-donating diethylamino (R^2^ = (C_2_H_5_)_2_N) at position 7′ (**2c**) or an electron-withdrawing Br substituent (R^1^ = Br) at position 6′ (**2d**) of the 2-iminocoumarin scaffold were shown to display a comparable level of ability to inhibit the growth of human cancer cell lines (IC_50_ values in the range of 2.19–8.15 µM and 1.21–6.97 µM, respectively). Interestingly, introducing an electron-donating methyl group (R^1^ = CH_3_) at position 6′ resulted in slight decrease in antiproliferative potency (compare **2e** with **2c** and **2d**: IC_50_ = 3.60–11.17 µM vs. 2.19–8.15 µM and 1.21–6.97 µM).

The above pattern was also seen in the series of 4-phenylpiperazine-containing complexes **2f**-**h**. Thus, replacement of the 7′-diethylamino group (R^2^ = (C_2_H_5_)_2_N) for an electron-withdrawing 6′-Cl substituent (R^1^ = Cl) in the 2-iminocoumarin scaffold led to compound with similar activity (compare **2g** with **2h**: IC_50_ = 1.21–1.66 µM vs. 1.97–3.28 µM). It should be pointed out that the lack of the substituents R^1^ and R^2^ yielded less active analogue **2f** (IC_50_ = 6.29–8.84 µM) (Table 1, Figure 10), but this compound still retained pronounced growth inhibitory properties toward the tested tumor cell lines. The results obtained seem to indicate that electronic and steric effects brought about the R^1^ and R^2^ substituents and may affect the biological activity of these compounds.

As in the case of copper(II), complexes of 2-iminocoumarins containing 1,3,5-triazine moiety, copper(II) complexes of benzoxazole/benzothiazole-2-iminocoumarins **4b**, **6a**-**c** were found to possess similar or higher activity than their free ligands **L3b** and **L5a-c**, respectively (IC_50_ = 0.04–13.42 µM vs. 0.06–14.03 µM) (Table 2). The exception to this was benzoxazole-containing compounds **4a** and **L3a**, where the complex **4a** was less active than its free ligand **L3a** (IC_50_ = 2.25–13.50 µM vs. 0.99–3.22 µM). The most active compound in this category was the 7′-ethylamino substituted benzoxazole-2-iminocoumarin complex **4b** which showed greater potency than cisplatin against the LCLC-103H, SISO and RT-4 cell lines with IC_50_ values ranging from 0.04 µM to 0.08 µM (Table 2, Figure 11).

On the other hand, analogue **6b** with benzothiazole instead a benzoxazole was found to be less active than the benzoxazole counterpart **4b** (IC_50_ = 1.18–1.96 µM vs. 0.04–0.08 µM), while benzothiazole-containing complex **6a** with a fluorine atom at position 6′ of the 2-iminocoumarin moiety showed higher potency than its benzoxazole analogue **4a** (IC_50_ = 0.13–2.08 µM vs. 2.25–13.50 µM) (Table 2, Figure 11). These observations are in line with our previous studies of free ligands—benzoxazole/benzothiazole-2-iminocoumarins [66].

Moreover, from the pattern of IC_50_ values, it can be concluded that within series of Cu(II) complexes of 2-imino-2*H*-chromen-3-yl-1,3,5-triazines and benzoxazole/benzothiazole-2*H*-chromen-2-imines, the best antiproliferative potency was shown for compounds bearing an electron-donating diethylamino substituent at position 7′ of the 2-iminocoumarin ring as evidenced by compounds **2g** (Table 1, Figure 10) and **4b** (Table 2, Figure 11), nonetheless benzoxazole-containing complex **4b** was significantly more active than 2-iminocoumarin-1,3,5-triazine complex **2g** in the LCLC-103H, SISO, RT-4 cell lines (IC_50_ = 0.04–0.08 µM vs. 1.21–1.66 µM). Additionally, the data presented above support the hypothesis that by introducing a metal ion into an organic moiety, the antiproliferative activity may be enhanced [10,11].

## 3. Experimental Section

### 3.1. General Information

The melting points were determined with a Boëtius apparatus and are uncorrected. The infrared spectra were recorded on a Nicolet 380 FT-IR spectrophotometer (Thermo Fisher Scientific Inc., Waltham, MA, USA). The elemental analyses of carbon, hydrogen, nitrogen, and sulfur determined for compounds were within ± 0.4% of the theoretical values.

UV-Vis spectra were recorded on Carl Zeiss Technology Spekol 1200 (Analytik Jena AG, Jena, Germany) in a 1.0 cm cuvette maintained at 37 °C by a thermostatically controlled cuvette holder.

Diffraction experiments were carried out at room temperature with an Oxford Diffraction SuperNova diffractometer (Agilent Technologies Inc., Santa Clara, CA, USA) using Cu Kα radiation. Diffraction data were processed with CrysAlisPro software [68]. The structures were solved with the program SHELXT [69] and refined by full-matrix least-squares method on *F*^2^ with SHELXL-2018/3 [70] within the Olex2 software [71]. C-H hydrogen atoms were placed in calculated positions and refined as riding on their carriers. In **2f**, **2h** and **6c** hydrogen atoms from the N-H groups were freely refined, whereas those in **2g** were placed in the geometrically predicted positions and refined as riding on their carriers.

Molecular modeling studies were performed at ab initio level using the density functional method (B3LYP) with the 6-31G* basis set as implemented into SPARTAN program version ‘14 V 1.1.4’ [72].

### 3.2. Chemistry

#### 3.2.1. Synthesis of Copper(II) Complexes of 2-imino-2*H*-chromen-3-yl-1,3,5-triazine derivatives **2a** and **2d** (Method A)

A solution of appropriate 2-iminocoumarin-1,3,5-triazine derivative **L1a** or **L1d** (1 mmol) in dimethylformamide (2–4 mL) containing 1–2% of water was heated to a tem-perature of 80 °C and then cooled. Copper(II) chloride dihydrate (3 mmol) was dissolved in 1 mL of dimethylformamide and added gradually to the solution of 1,3,5-triazine in dimethylformamide at room temperature (20–22°C). Upon slow evaporation of the solvent over 1–4 days, copper(II) complex was formed. Precipitate was filtered, washed with cold dimethylformamide (2 × 0.5 mL) and dried in a desiccator. The following complexes were obtained to above procedure:

*Dichloro[4-(2-imino-2H-chromen-3-yl)-6-(piperidin-1-yl)-1,3,5-triazin-2-amine]copper(II)* (2a). Yield 13% as orange colored powder; m.p. 261–262 °C; IR (KBr) ν_max_ (cm^−1^): 3468, 2934, 2855, 1647, 1550, 1482, 1240, 803, 776. Anal. calcd for C_17_H_18_Cl_2_CuN_6_O (456.82): C, 44.70; H, 3.97; N, 18.40. Found: C, 44.62; H, 3.78; N, 18.32.

*Dichloro[4-(6-bromo-2-imino-2H-chromen-3-yl)-6-(3,5,5-trimethyl-4,5-dihydro-1H-pyrazol-1-yl)-1,3,5-triazin-2-amine]copper(II)* (2d). Yield 23% as green crystals; m.p. 211–212 °C; IR (KBr) ν_max_ (cm^−1^): 3298, 2923, 1643, 1544, 1493, 1233, 804, 756. Anal. calcd for C_18_H_18_BrCl_2_CuN_7_O (562.74): C, 38.42; H, 3.22; N, 17.42. Found: C, 38.36; H, 3.12; N, 17.23.

#### 3.2.2. Synthesis of Copper(II) Complexes of 2-imino-2*H*-chromen-3-yl-1,3,5-triazine Derivatives **2b-c,** and **2f** (Method B)

An appropriate 2-iminocoumarin-1,3,5-triazine derivative L1b, L1c, or L1f (1 mmol) was dissolved in 98% ethanol (4–10 mL) at a temperature of 40 °C. After cooling to room temperature (20–22 °C), copper(II) chloride dihydrate (3 mmol) dissolved in 1 mL of 98% ethanol and was added gradually. If precipitate of 1,3,5-triazine derivative was formed after cooling to room temperature the second portion of 98% ethanol was added. Upon slow evaporation of the solvent over 1–4 days, the copper(II) complex was formed. Precipitate was filtered off, washed with cold 98% ethanol (2 × 0.5 mL) and dried in a desiccator. The following complexes were obtained to above procedure:

*Dichloro{4-[7-(diethylamino)-2-imino-2H-chromen-3-yl]-6-morpholino-1,3,5-triazin-2-amine}copper(II)* (2b). Yield 26% as violet crystals; m.p. 189–190 °C; IR (KBr) ν_max_ (cm^−1^): 3307, 1653, 1598, 1498, 1351, 1237, 1173, 800. Anal. Calcd for C_20_H_25_Cl_2_CuN_7_O_2_ (529.91): C, 45.33; H, 4.76; N, 18.50. Found: C, 45.14; H, 4.68; N, 18.42.

*Dichloro{4-[7-(diethylamino)-2-imino-2H-chromen-3-yl]-6-(3,5,5-trimethyl-4,5-dihydro-1H-pyrazol-1-yl)-1,3,5-triazin-2-amine}copper(II)* (2c). Yield 18% as dark brown crystals; m.p. 189 °C (dec.); IR (KBr) ν_max_ (cm^−1^): 3110, 1654, 1595, 1500, 1466, 1340, 1247, 1183, 1015, 800, 693. Anal. calcd for C_22_H_28_Cl_2_CuN_8_O (554.96): C, 47.61; H, 5.09; N, 20.19. Found: C, 47.58; H, 5.01; N, 20.08.

*Tetrachloro-bis-{[4-(2-imino-2H-chromen-3-yl)-6-(4-phenylpiperazin-1-yl)-1,3,5-triazin-2-amine]copper(II)}* (2f). Yield 23% as dark green crystals; m.p. 169–170 °C; IR (KBr) ν_max_ (cm^−1^): 3489, 3336, 1649, 1546, 1494, 1231, 804, 757.

Crystal data for **2f** ([(C_22_H_21_N_7_O)_2_Cl_4_Cu_2_]·C_2_H_5_OH, *M* = 1113.86 g/mol): triclinic, space group P-1 (no. 2), *a* = 10.4108(4) Å, *b* = 11.3726(4) Å, *c* = 11.4595(5) Å, *α* = 83.833(3)°, *β* = 64.171(4)°, *γ* = 77.430(3)°, *V* = 1191.84(9) Å^3^, *Z* = 1, *T* = 293(2) K, μ(Cu Kα) = 3.641 mm^−1^, *Dcalc* = 1.552 g/cm^3^, 24,433 reflections measured (8.574° ≤ 2Θ ≤ 151.718°), 4866 unique (*R*_int_ = 0.0381, *R*_sigma_ = 0.0196) which were used in all calculations. The final *R*_1_ was 0.0393 (I > 2σ(I)) and *wR*_2_ was 0.1187 (all data). The crystals were unstable in the air. The solvent ethanol molecule is disordered around an inversion center. The methylene and methyl group C atoms from the ethanol molecules occupying different sites were found in overlapping positions. The molecule of **2f** is shown in Figure 4.

#### 3.2.3. Synthesis of Copper(II) Complexes of 2-imino-2*H*-chromen-3-yl-1,3,5-triazine Derivatives **2e**, **2g** and **2h** (Method C)

An appropriate 2-iminocoumarin-1,3,5-triazine derivative L1e, L1g or L1h (1 mmol) was dissolved in methanol (6 mL) at a temperature of 40 °C. After cooling to room temperature (20–22 °C) copper(II) chloride dihydrate (3 mmol) dissolved in 1 mL of methanol and was added gradually. Upon slow evaporation of the solvent over 1–4 days, copper(II) complex was formed. Precipitate was filtered off, washed with cold methanol (2 × 0.5 mL) and dried in a desiccator. The following complexes were obtained to above procedure:

*Dichloro[4-(2-imino-6-methyl-2H-chromen-3-yl)-6-(3,5,5-trimethyl-4,5-dihydro-1H-pyrazol-1-yl)-1,3,5-triazin-2-amine]copper(II)* (2e). Yield 21% as green crystals; m.p. 171–172 °C; IR (KBr) ν_max_ (cm^−1^): 3227, 2923, 1654, 1582, 1508, 1467, 1380, 1340, 1250, 803. Anal. calcd for C_19_H_21_Cl_2_CuN_7_O (497.87): C, 45.84; H, 4.25; N, 19.69. Found: C, 45.68; H, 4.12; N, 19.54.

*Dichloro{4-[7-(diethylamino)-2-imino-2H-chromen-3-yl]-6-(4-phenylpiperazin-1-yl)-1,3,5-triazin-2-amine}copper(II)* (2g). Yield 27% as violet crystals; m.p. 194–195 °C; IR (KBr) ν_max_ (cm^−1^): 3352, 2923, 1654, 1586, 1501, 1232, 804, 748.

Crystal data for **2g** ([(C_26_H_30_N_8_OCl_2_Cu], *M* = 605.02 g/mol): triclinic, space group P-1 (no. 2), *a* = 8.8366(7) Å, *b* = 11.1171(8) Å, *c* = 15.0104(9) Å, *α* = 100.138(6)°, *β* = 93.417(6)°, *γ* = 105.259(7)°, *V* = 1391.65(18) Å^3^, *Z* = 2, *T* = 293(2) K, μ(Cu Kα) = 3.161 mm^−1^, *Dcalc* = 1.444 g/cm^3^, 14,878 reflections measured (6.018° ≤ 2Θ ≤ 133.202°), 4927 unique (*R*_int_ = 0.0242, *R*_sigma_ = 0.0211) which were used in all calculations. The final *R*_1_ was 0.0423 (I > 2σ(I)) and *wR*_2_ was 0.1293 (all data). One of the ethyl groups of the diethylamino substituent is disordered over two sites. The molecule of **2g** is shown in Figure 5.

*Dichloro[4-(6-chloro-2-imino-2H-chromen-3-yl)-6-(4-phenylpiperazin-1-yl)-1,3,5-triazin-2-amine]copper(II)* (2h). Yield 22% as greenish crystals; m.p. 191 °C (dec.); IR (KBr) ν_max_ (cm^−1^): 3295, 1643, 1570, 1545, 1496, 1233, 803, 761.

Crystal data for **2h** ([(C_22_H_20_ClN_7_O]Cl_2_Cu]·0.5(H_2_O), *M* = 577.35g/mol): monoclinic, space group P2_1_/n (no. 14), *a* = 16.9813(4) Å, *b* = 8.8458(2) Å, *c* = 17.1929(4) Å, *β* = 109.043(3)°, *V* = 2441.26(11) Å^3^, *Z* = 4, *T* = 294 K, μ(Cu Kα) = 4.563 mm^−1^, *Dcalc* = 1.571 g/cm^3^, 13,722 reflections measured (6.352° ≤ 2Θ ≤ 133.198°), 4315 unique (*R*_int_ = 0.030 0, *R*_sigma_ = 0.0224) which were used in all calculations. The final *R*_1_ was 0.0453 (I > 2σ(I)) and *wR*_2_ was 0.1335 (all data). The water molecule is disordered over two sites related by an inversion center. Hydrogen atoms of the water molecule were not located. The molecule of **2h** is shown in Figure 6.

#### 3.2.4. Synthesis of Copper(II) Complexes of 3-(benzoxazol-2-yl)-2*H*-chromen-2-imine Derivatives **4a-b** (General Procedure)

An appropriate 3-(benzoxazol-2-yl)-2*H*-chromen-2-imine derivative **L3a** or **L3b** (1 mmol) was dissolved in 98% ethanol (4–10 mL) at a temperature of 40 °C. To the solution was added dropwise at ambient temperature (20–22 °C) copper(II) chloride dihydrate (3 mmol) dissolved in ethanol (1 mL). The solution was left at room temperature (20–22 °C) and the solvent was slowly evaporated. The resulting precipitate (24 h to 4 days) was filtered, washed with cold ethanol (2 × 0.5 mL), and dried in a desiccator. The following complexes were prepared according to above given procedure.

*Dichloro[3-(benzo[d]oxazol-2-yl)-6-fluoro-2H-chromen-2-imine]copper(II)* (**4a**). Yield 20% as violet-brown crystals; m.p. > 350 °C; IR (KBr) ν_max_ (cm^−1^)**:** 3119, 3067, 2927, 1635, 1586, 1560, 1471, 1430, 1374, 1269, 1205, 1155, 1054, 876, 794, 722. Anal. calcd for C_16_H_9_Cl_2_CuFN_2_O_2_ (414.71): C, 46.34; H, 2.19; N, 6.76. Found: C, 46.17; H, 2.35; N, 6.85.

*Dichloro[3-(benzo[d]oxazol-2-yl)-N,N-diethyl-2-imino-2H-chromen-7-amine]copper(II)* (**4b**). Yield 24% as violet crystals; m.p. 229–230 °C; IR (KBr) ν_max_ (cm^−1^): 3292, 2976, 1654, 1601, 1574, 1527, 1497, 1399, 1343, 1240, 1184, 1123, 1077, 973, 773, 757. Anal. calcd for C_20_H_19_Cl_2_CuN_3_O_2_ (467.84): C, 51.35; H, 4.09; N, 8.98. Found: C, 51.43; H, 3.89; N, 9.05.

#### 3.2.5. Synthesis of Copper(II) Complexes of 3-(benzothiazol-2-yl)-2*H*-chromen-2-imine Derivatives **6a-c** (General Procedure)

An appropriate 3-(benzothiazol-2-yl)-2*H*-chromen-2-imine derivative **L5a**, **L5b**, or **L5c** (1 mmol) was dissolved in dimethylformamide (2–4 mL) containing 1–2% water at a temperature of 80 °C. To the resulting solution, copper(II) chloride dihydrate (3 mmol) was added dropwise at ambient temperature (20–22 °C) and dissolved in dimethylformamide (1 mL). The solution was left at room temperature (20–22 °C) and the solvent was slowly evaporated. The resulting precipitate (24 h to 4 days) was filtered, washed with cold dimethylformamide (2 × 0.5 mL) and dried in a desiccator. The following complexes were prepared according to above given procedure.

*Dichloro[3-(benzo[d]thiazol-2-yl)-6-fluoro-2H-chromen-2-imine]copper(II)* (**6a**). Yield 29% as violet-dark blue crystals; m.p. 231–232 °C; IR (KBr) ν_max_ (cm^−1^): 3178, 3062, 3017, 1664, 1642, 1596, 1566, 1491, 1413, 1272, 1248, 957, 880, 784, 763, 753. Anal. calcd for C_16_H_9_Cl_2_CuFN_2_OS (430.77): C, 44.61; H, 2.11; N, 6.50. Found: C, 44.49; H, 2.31; N, 5.87.

*Dichloro[3-(benzo[d]thiazol-2-yl)-N,N-diethyl-2-imino-2H-chromen-7-amine]copper(II)* (**6b**). Yield 29% as violet-dark blue crystals; m.p. 231–232 °C; IR (KBr) ν_max_ (cm^−1^): 3338, 2963, 2922, 1646, 1592, 1573, 1510, 1380, 1350, 1271, 1185, 1115, 756, 745. Anal. calcd for C_20_H_19_Cl_2_CuN_3_OS (483.90): C, 49.64; H, 3.96; N, 8.63. Found: C, 49.55; H, 3.83; N, 8.57.

*Dichloro[3-(benzo[d]thiazol-2-yl)-6-methyl-2H-chromen-2-imine]copper(II)* (**6c**). Yield 24% as dark green crystals; m.p. 248–249 °C; IR (KBr) ν_max_ (cm^−1^): 3190, 3007, 2922, 1636, 1613, 1588, 1568, 1457, 1412, 1327, 1247, 1209, 1127, 956, 861, 825, 752.

Crystal data for **6c** ([(C_17_H_12_N_2_OS)Cl_2_Cu]·C_3_H_7_NO, *M* = 499.88 g/mol): triclinic, space group P-1 (no. 2), *a* = 8.8247(3) Å, *b* = 9.9302(4) Å, *c* = 12.6944(5) Å, *α* = 104.339(3)°, *β* = 90.641(3)°, *γ* = 102.895(3)°, *V* = 1047.99(7) Å^3^, *Z* = 2, *T* = 293(2) K, μ(Cu Kα) = 4.935 mm^−1^, *Dcalc* = 1.584 g/cm^3^, 8178 reflections measured (7.206° ≤ 2Θ ≤ 136.502°), 3828 unique (*R*_int_ = 0.0181, *R*_sigma_ = 0.0200) which were used in all calculations. The final *R*_1_ was 0.0266 (I > 2σ(I)) and *wR*_2_ was 0.0770 (all data). The asymmetric unit of **6c** is shown in Figure 7.

### 3.3. Stability Studies

To 5.0 mL of phosphate-buffered saline at pH 7.4 and pre-warmed at 37 °C was added 10 µL of a 20 mM DMF solution of the copper(II) complex **2a**-**h**, **4a**-**b**, or **6a**-**c**, resulting in a final concentration of 40 µM. The solution was then transferred to a 1.0 cm quartz cuvette and placed in a heated cuvette holder maintained at 37 °C. UV-Vis spectra were recorded at 60 min intervals between wavelengths of 200 nm and 600 nm by means of UV-Vis spectrophotometer (Carl Zeiss Technology Spekol 1200, Analytik Jena AG, Jena, Germany) connected to a personal computer running the *Vision 32* software.

### 3.4. In Vitro Cytotoxicity Studies

The antiproliferative activities of the compounds on human cancer cells were determined by the Crystal Violet assay as previously described [67]. All cell culture materials were purchased from SigmaAldrich (Deisenhofen, FRG). Cancer cell lines: human pancreas cell adenocarcinoma (DAN-G), human lung carcinoma (A-427), human non-small cell lung carcinoma (LCLC-103H), human uterine cervical adenocarcinoma (SISO), and human urinary bladder carcinoma (RT-4) and were obtained from the German Collection of Microorganisms and Cell Cultures (DSMZ, Deutsche Sammlung von Mikroorganismen und Zellkulturen, Braunschweig, FRG). The culture medium for cell lines was RPMI-1640 medium containing 2 g/L HCO_3_, and 10% sterile calf serum, suitable for cell culture. Cells were incubated in a humid atmosphere of 5% CO_2_ at 37 °C in 75 cm^2^ plastic culture flasks (Sarstedt, Nümbrecht, FRG) and were passaged shortly before becoming confluent. For the cytotoxicity studies, 100 μL of a cell suspension was seeded into 96-well microtiter plates (Sarstedt) at a density of 1000 cells per well except for the LCLC-103H cell line, which was plated out at 250 cells per well. One day after plating, the cells were treated with a test substance at five concentrations per compound. The 1000-fold concentrated stock solutions in dimethylformamide (DMF) or dimethylsulfoxide (DMSO) were serially diluted by 50% in DMF or DMSO to give the feed solutions, which were diluted 500-fold into the culture medium. The controls received DMF or DMSO at a final concentration of 0.1%. Each concentration was tested in eight wells, with each well receiving 100 μL of the medium containing the substance. The concentration ranges were chosen to bracket the expected IC_50_ values as best as possible. Cells were then incubated for 96 h, after which time the medium was removed and replaced with 1% glutaraldehyde/phosphate-buffered saline for 30 min. The glutaraldehyde solution was discarded, and the plates washed with water before staining cells for 30 min with an aqueous solution of 0.02% Crystal Violet. After washing out excess Crystal Violet from the plates, optical density (OD) was measured at λ = 570 nm by use of an Anthos 2010 plate reader (Salzburg, Austria). Corrected T/C values were calculated according to the Equation (1):

(T/C)_corr_(%) = (O.D.T − O.D.c.0)/(O.D.C − O.D.c.0) × 100 (1)

where O.D.T is the mean optical density of the wells from the treated cells; O.D.C the mean optical density of the control wells and O.D.c.0 the mean optical density on day 0 when the time drug was added. The IC_50_ values were estimated by linear least-squares regression of the (T/C)_corr_ values versus the logarithm of the substance concentration by using MS-Excel; only concentrations that yielded (T/C)_corr_ values between 10% and 90% were used in the calculation of the IC_50_ values. The reported IC_50_ values are the averages of three independent experiments [67].

## 4. Conclusions

In this paper we have described the synthesis, structure, and cytotoxic activity of two series of novel Cu(II) complexes of 2-iminocoumarins bearing a 1,3,5-triazine or benzoxazole/benzothiazole moiety. The coordination of 2-iminocoumarin ligands with copper(II) results in metal complexes with significant growth inhibitory properties toward tested human tumor cell lines with IC_50_ values ranging from 0.04 to 15.66 μM. Furthermore, it was observed that newly prepared Cu(II) complexes often possess higher activity than the related ligands. As expected, in the series of Cu(II) complexes of 2-imino-2*H*-chromen-3-yl-1,3,5-triazine derivatives, the highest potency was shown for compound **2g** bearing 4-phenylpiperazine moiety at position 6 of the 1,3,5-triazine ring and an electron-donating diethylamino group at position 7’ of the 2-iminocoumarin scaffold. On the other hand, the SAR studies of this series indicate that 2-iminocoumarin scaffold is open for variation such that halogen, diethylamino, and methyl substituents are allowed. In turn, in the category of Cu(II) complexes of benzoxazole/benzothiazole-2*H*-chromen-2-imines the best antiproliferative activity was found for 7’-diethylamino-substituted benzoxazole derivative **4b**. Additionally, compound **4b** was found to be the most promising agent with higher potency than cisplatin against the LCLC-103H, SISO, and RT-4 cell lines. Based on the results obtained, one may conclude that the most active compounds in either triazine or benzazole series possess a diethylamino group in the 2-iminocoumarin skeleton as evidenced by compounds **2g** and **4b**, suggesting that this group is somehow involved in the mechanism of action. In this context, it is worth noting that coumarin-based agents [65,73,74,75,76] display the intercalative mode of binding properties with DNA and are well-known DNA minor groove binders. Thus, the presence of diethylamino group in the structure of designed compounds may increase the efficiency in the intercalative binding giving extra non-covalent force between the substituent and DNA grooves. Therefore, additional work is needed to elucidate their mechanism of action.

## Figures and Tables

**Table 1:** IC_50_ (µM) values of copper(II) complexes of 2-imino-2*H*-chromen-3-yl-1,3,5-triazines **2a-h**, their corresponding ligands **L1a**-**h** and reference compound in five human cancer cell lines after 96 h **^a^**.

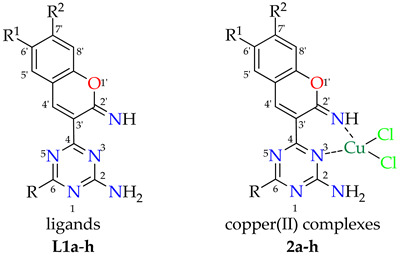								
***Compd*:** **Complex** (ligand)	**R**	**R^1^**	**R^2^**	***Cell Line***				
				**DAN**-**G**	**A-427**	**L** **CLC-103H**	**SISO**	**RT**-**4**
**2a** (**L1a**)	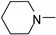	H	H	10.60 ± 1.24 (15.12) **^a^**	10.69 ± 1.19 (9.04)	10.13 ± 3.55 (14.17)	15.66 ± 4.28 (21.33)	1.82 ± 0.75 (16.24)
**2b** (**L1b**)	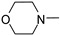	H	(C_2_H_5_)_2_N	2.34 ± 0.12 (9.21)	12.97 ± 2.11 (7.62)	8.02 ± 2.80 (6.18)	6.14 ± 0.21 (5.67)	*
**2c** (**L1c**)	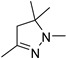	H	(C_2_H_5_)_2_N	8.15 ± 0.28 (11.19)	2.19 ± 0.54 (8.16)	*	7.59 ± 0.53 (11.64)	7.17 ± 0.72 (11.04)
**2d** (**L1d**)	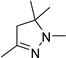	Br	H	*	*	6.97 ± 3.18 (27.42)	6.94 ± 0.48 (7.69)	1.21 ± 1.41 (21.40)
**2e** (**L1e**)	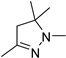	CH_3_	H	6.76 ± 0.84 (11.91)	3.60 ± 0.59 (13.56)	11.14 ± 1.83 (15.47)	7.33 ± 0.31 (14.44)	11.17 ± 1.78 (6.91)
**2f** (**L1f**)	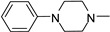	H	H	8.73 ± 0.62 (8.35)	6.29 ± 0.46 (14.79)	8.84 ± 3.57 (21.12)	12.99 ± 1.18 (15.24)	7.05 ± 1.22 (16.24)
**2g** (**L1g**)	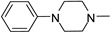	H	(C_2_H_5_)_2_N	1.61 ± 0.09 (2.14)	1.31 ± 0.37 (1.51)	1.66 ± 0.07 (2.21)	*	1.21 ± 0.07 (1.66)
**2h** (**L1h**)	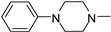	Cl	H	3.28 ± 0.33 (23.26)	2.33 ± 0.42 (37.19)	2.77 ± 1.09 (34.24)	4.09 ± 0.23 (29.86)	1.97 ± 0.94 (26.32)
**CDDP ^b^**				0.73 ± 0.34	1.96 ± 0.54	0.90 ± 0.19	0.24 ± 0.06	1.61 ± 0.16

**^a^** values shown are mean ± SD from three independent experiments, in parentheses the corresponding values for ligands **L1a-h** previously described in ref. [65]. **^b^** cisplatin—ref. [67]. * IC_50_ values could not be determined with the required precision.

**Table 2:** IC_50_ (µM) values of copper(II) complexes of 3-(benzoxazol-2-yl)-2*H*-chromen-2-imines **4a**-**b**, and 3-(benzothiazol-2-yl)-2*H*-chromen-2-imines **6a**-**c**, their corresponding ligands **L3a-b**, **L5a-b,** and reference compounds in five human cancer cell lines after 96 h **^a^**.

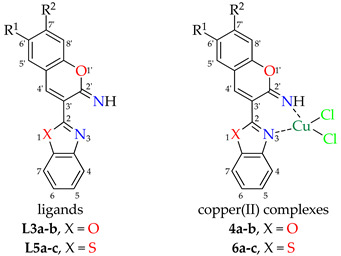							
***Compd*:** **Complex** (ligand)	**R^1^**	**R^2^**	***Cell Line***				
			**DAN**-**G**	**A-427**	**L** **CLC-103H**	**SISO**	**RT**-**4**
**4a** (**L3a**)	F	H	13.50 ± 1.8 (1.96) **^a^**	2.25 ± 0.12 (2.38)	2.35 ± 0.26 (0.99)	12.72 ± 8.41 (2.44)	2.76 ± 0.35 (3.22)
**4b** (**L3b**)	H	(C_2_H_5_)_2_N	*	*	0.04 ± 0.01 (0.06)	0.08 ± 0.01 (0.29)	0.08 ± 0.02 (0.27)
**6a** (**L5a**)	F	H	1.56 ± 0.01 (1.45)	*	0.13 ± 0.01 (0.98)	1.45 ± 0.19 (1.12)	2.08 ± 0.28 (1.11)
**6b** (**L5b**)	H	(C_2_H_5_)_2_N	1.18 ± 0.09 (1.05)	1.34 ± 0.29 (1.22)	*	1.33 ± 0.01 (1.13)	1.96 ± 0.05 (0.54)
**6c** (**L5c**)	CH_3_	H	12.99 ± 2.65 (12.74)	13.42 ± 1.34 (13.05)	12.43 ± 3.43 (13.67)	13.07 ± 1.76 (13.14)	10.75 ± 1.84 (14.03)
**CDDP ^b^**			0.73 ± 0.34	1.96 ± 0.54	0.90 ± 0.19	0.24 ± 0.06	1.61 ± 0.16

**^a^** values shown are mean ± SD from three independent experiments, in parentheses the corresponding values for ligands **L3a-b** and **L5a-c** previously described in ref. [66]. **^b^** cisplatin—ref. [67]. * IC_50_ values could not be determined with the required precision.

## Data Availability

Not applicable.

## Supplementary Materials

The following supporting information can be downloaded at: https://www.mdpi.com/article/10.3390/molecules27217155/s1, checkCIF/PLATON reports for compounds **2h**, **2f**, **2g** and **6c**. Crystallographic data (CCDC 2191576-2191579) associated with this article are available online at: www.ccdc.cam.ac.uk/conts/retrieving.html (accessed on 1 September 2022).

## Sample Availability

Samples of the compounds **L1a**-**h**, **2a**-**h**, **L3a**-**b**, **4a**-**b**, **L5a**-**c** and **6a**-**c** are not available from the authors.
