# Supplementary material for: Synthesis, Structure and Cytotoxic Properties of Copper(II) Complexes of 2-Iminocoumarins Bearing a 1,3,5-Triazine or Benzoxazole/Benzothiazole Moiety
Source: Molecules. 2022 Oct 22;27(21):7155. doi: 10.3390/molecules27217155 (PMC9659224; doi:10.3390/molecules27217155)

checkCIF/PLATON report

Structure factors have been supplied for datablock(s) sac150

THIS REPORT IS FOR GUIDANCE ONLY. IF USED AS PART OF A REVIEW PROCEDURE FOR PUBLICATION, IT SHOULD NOT REPLACE THE EXPERTISE OF AN EXPERIENCED CRYSTALLOGRAPHIC REFEREE.

No syntax errors found. CIF dictionary Interpreting this report

Datablock: sac150

Bond precision: C-C = 0.0045 A Wavelength=1.54184

Cell:	a=10.4108(4)	b=11.3726(4)	c=11.4595(5)
	alpha=83.833(3)	beta=64.171(4)	gamma=77.430(3)
Temperature:	293 K		

	Calculated	Reported
Volume	1191.84(9)	1191.84(9)
Space group	P -1	P -1
Hall group	-P 1	-P 1
Moiety formula	C44 H42 Cl4 Cu2 N14 O2, C2 H6 O	C44 H42 Cl4 Cu2 N14 O2, C2 H6 O
Sum formula	C46 H48 Cl4 Cu2 N14 O3	C46 H48 Cl4 Cu2 N14 O3
Mr	1113.88	1113.86
Dx,g cm-3	1.552	1.552
Z	1	1
Mu (mm-1)	3.641	3.641
F000	572.0	572.0
F000'	570.82	
h,k,lmax	13,14,14	13,14,14
Nref	4976	4866
Tmin,Tmax	0.438,0.483	0.485,1.000
Tmin'	0.319	

Correction method= # Reported T Limits: Tmin=0.485 Tmax=1.000
AbsCorr = MULTI-SCAN

Data completeness= 0.978 Theta(max)= 75.859

R(reflections)= 0.0393(4407) wR2(reflections)= 0.1186(4866)

S = 1.072 Npar= 329

The following ALERTS were generated. Each ALERT has the format
test-name_ALERT_alert-type_alert-level.
Click on the hyperlinks for more details of the test.

Alert level C

```
PLAT260_ALERT_2_C Large Average Ueq of Residue Including      O1EA      0.147 Check
PLAT352_ALERT_3_C Short  N-H (X0.87,N1.01A)  N11      - H11      .       0.75 Ang.
PLAT360_ALERT_2_C Short  C(sp3)-C(sp3) Bond  C1EA     - C1EA_b   .       1.43 Ang.
PLAT410_ALERT_2_C Short Intra H...H Contact  H21A     ..H26      .       1.92 Ang.
                                                      x,y,z  =      1_555 Check
PLAT410_ALERT_2_C Short Intra H...H Contact  H23B     ..H30      .       1.99 Ang.
                                                      x,y,z  =      1_555 Check
PLAT420_ALERT_2_C D-H Bond Without Acceptor  N11      --H11      .     Please Check
PLAT911_ALERT_3_C Missing FCF Refl Between Thmin & STh/L=    0.600         24 Report
PLAT913_ALERT_3_C Missing # of Very Strong Reflections in FCF ....          9 Note
```

Alert level G

```
PLAT007_ALERT_5_G Number of Unrefined Donor-H Atoms .............           1 Report
PLAT012_ALERT_1_G No       _shelx_res_checksum Found in CIF ......     Please Check
PLAT199_ALERT_1_G Reported _cell_measurement_temperature ..... (K)        293 Check
PLAT200_ALERT_1_G Reported   _diffrn_ambient_temperature ..... (K)        293 Check
PLAT232_ALERT_2_G Hirshfeld Test Diff (M-X)  Cu1      --Cl2_a    .        6.0 s.u.
PLAT300_ALERT_4_G Atom Site Occupancy of O1EA       Constrained at        0.5 Check
PLAT300_ALERT_4_G Atom Site Occupancy of H1EA       Constrained at        0.5 Check
PLAT300_ALERT_4_G Atom Site Occupancy of H1ED       Constrained at        0.5 Check
PLAT302_ALERT_4_G Anion/Solvent/Minor-Residue Disorder (Resd  2  )        33% Note
PLAT720_ALERT_4_G Number of Unusual/Non-Standard Labels .........           6 Note
PLAT779_ALERT_4_G Suspect or Irrelevant (Bond) Angle(s) in CIF ...       5.90 Deg.
              O1EA -C1EA -H1EA    1_555   1_555   1_555 ......     #  117 Check
PLAT789_ALERT_4_G Atoms with Negative _atom_site_disorder_group  #          1 Check
PLAT794_ALERT_5_G Tentative Bond Valency for Cu1       (II)      .       2.10 Info
PLAT804_ALERT_5_G Number of ARU-Code Packing Problem(s) in PLATON           1 Info
PLAT910_ALERT_3_G Missing # of FCF Reflection(s) Below Theta(Min).          1 Note
PLAT912_ALERT_4_G Missing # of FCF Reflections Above STh/L=  0.600         86 Note
PLAT978_ALERT_2_G Number C-C Bonds with Positive Residual Density.          6 Info
```

---

0 **ALERT level A** = Most likely a serious problem - resolve or explain
0 **ALERT level B** = A potentially serious problem, consider carefully
8 **ALERT level C** = Check. Ensure it is not caused by an omission or oversight
17 **ALERT level G** = General information/check it is not something unexpected

3 ALERT type 1 CIF construction/syntax error, inconsistent or missing data
7 ALERT type 2 Indicator that the structure model may be wrong or deficient
4 ALERT type 3 Indicator that the structure quality may be low
8 ALERT type 4 Improvement, methodology, query or suggestion
3 ALERT type 5 Informative message, check

It is advisable to attempt to resolve as many as possible of the alerts in all categories. Often the minor alerts point to easily fixed oversights, errors and omissions in your CIF or refinement strategy, so attention to these fine details can be worthwhile. In order to resolve some of the more serious problems it may be necessary to carry out additional measurements or structure refinements. However, the purpose of your study may justify the reported deviations and the more serious of these should normally be commented upon in the discussion or experimental section of a paper or in the "special_details" fields of the CIF. checkCIF was carefully designed to identify outliers and unusual parameters, but every test has its limitations and alerts that are not important in a particular case may appear. Conversely, the absence of alerts does not guarantee there are no aspects of the results needing attention. It is up to the individual to critically assess their own results and, if necessary, seek expert advice.

Publication of your CIF in IUCr journals

A basic structural check has been run on your CIF. These basic checks will be run on all CIFs submitted for publication in IUCr journals (*Acta Crystallographica*, *Journal of Applied Crystallography*, *Journal of Synchrotron Radiation*); however, if you intend to submit to *Acta Crystallographica Section C* or *E* or *IUCrData*, you should make sure that full publication checks are run on the final version of your CIF prior to submission.

Publication of your CIF in other journals

Please refer to the *Notes for Authors* of the relevant journal for any special instructions relating to CIF submission.

PLATON version of 18/05/2022; check.def file version of 17/05/2022

Datablock sac150 - ellipsoid plot

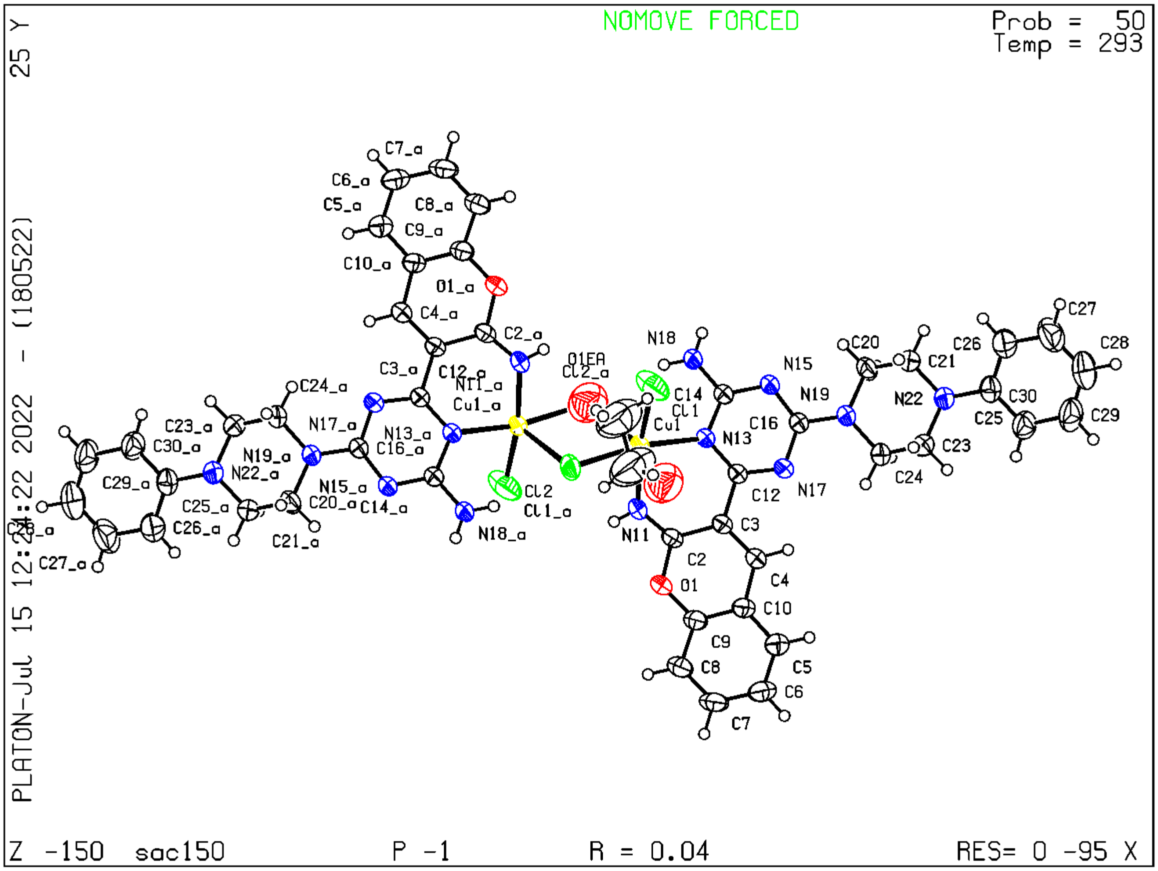

Supplement: Supplementary file 1 [file molecules-27-07155-s001.zip › checkcif-compound_2f_rev.pdf]
