# Supplementary material for: Synthesis, Structure and Cytotoxic Properties of Copper(II) Complexes of 2-Iminocoumarins Bearing a 1,3,5-Triazine or Benzoxazole/Benzothiazole Moiety
Source: Molecules. 2022 Oct 22;27(21):7155. doi: 10.3390/molecules27217155 (PMC9659224; doi:10.3390/molecules27217155)

checkCIF/PLATON report

Structure factors have been supplied for datablock(s) sac158

THIS REPORT IS FOR GUIDANCE ONLY. IF USED AS PART OF A REVIEW PROCEDURE FOR PUBLICATION, IT SHOULD NOT REPLACE THE EXPERTISE OF AN EXPERIENCED CRYSTALLOGRAPHIC REFEREE.

No syntax errors found. CIF dictionary Interpreting this report

Datablock: sac158

```
Bond precision:   C-C = 0.0045 A          Wavelength=1.54184

Cell:           a=8.8366(7)        b=11.1171(8)      c=15.0104(9)
                alpha=100.138(6)   beta=93.417(6)    gamma=105.259(7)
Temperature:    293 K

                 Calculated                   Reported
Volume           1391.65(18)                  1391.65(18)
Space group      P -1                         P -1
Hall group       -P 1                         -P 1
Moiety formula   C26 H30 Cl2 Cu N8 O          C26 H30 Cl2 Cu N8 O
Sum formula      C26 H30 Cl2 Cu N8 O          C26 H30 Cl2 Cu N8 O
Mr               605.03                       605.02
Dx,g cm-3        1.444                        1.444
Z                2                            2
Mu (mm-1)        3.161                        3.161
F000             626.0                        626.0
F000'            624.92
h,k,lmax         10,13,17                     10,13,17
Nref             4932                         4927
Tmin,Tmax        0.767,0.969                  0.764,1.000
Tmin'            0.483

Correction method= # Reported T Limits: Tmin=0.764 Tmax=1.000
AbsCorr = MULTI-SCAN

Data completeness= 0.999          Theta(max)= 66.601

R(reflections)= 0.0423( 3895)                        wR2(reflections)=
                                                     0.1293( 4927)
S = 1.039                    Npar= 366
```

The following ALERTS were generated. Each ALERT has the format
test-name_ALERT_alert-type_alert-level.
Click on the hyperlinks for more details of the test.

Alert level C

```
PLAT094_ALERT_2_C Ratio of Maximum / Minimum Residual Density ....       2.14 Report
PLAT220_ALERT_2_C NonSolvent   Resd 1  C   Ueq(max)/Ueq(min) Range        3.6 Ratio
PLAT222_ALERT_3_C NonSolvent Resd 1  H   Uiso(max)/Uiso(min) Range        4.6 Ratio
PLAT241_ALERT_2_C High   'MainMol' Ueq as Compared to Neighbors of        C29 Check
PLAT242_ALERT_2_C Low    'MainMol' Ueq as Compared to Neighbors of        C25 Check
PLAT420_ALERT_2_C D-H Bond Without Acceptor  N11      --H11      .     Please Check
PLAT601_ALERT_2_C Unit Cell Contains Solvent Accessible VOIDS of .         36 Ang**3
PLAT790_ALERT_4_C Centre of Gravity not Within Unit Cell: Resd.  #          1 Note
              C26 H30 Cl2 Cu N8 O
PLAT906_ALERT_3_C Large K Value in the Analysis of Variance ......      2.150 Check
PLAT911_ALERT_3_C Missing FCF Refl Between Thmin & STh/L=    0.595          5 Report
```

Alert level G

```
PLAT002_ALERT_2_G Number of Distance or Angle Restraints on AtSite          5 Note
PLAT003_ALERT_2_G Number of Uiso or Uij Restrained non-H Atoms ...          1 Report
PLAT007_ALERT_5_G Number of Unrefined Donor-H Atoms .............           3 Report
PLAT012_ALERT_1_G No       _shelx_res_checksum Found in CIF ......     Please Check
PLAT172_ALERT_4_G The CIF-Embedded .res File Contains DFIX Records          3 Report
PLAT186_ALERT_4_G The CIF-Embedded .res File Contains ISOR Records          1 Report
PLAT199_ALERT_1_G Reported _cell_measurement_temperature ..... (K)        293 Check
PLAT200_ALERT_1_G Reported   _diffrn_ambient_temperature ..... (K)        293 Check
PLAT301_ALERT_3_G Main Residue  Disorder ..............(Resd  1  )         5% Note
PLAT410_ALERT_2_G Short Intra H...H Contact  H8       ..H34D     .       2.09 Ang.
                                                       x,y,z  =      1_555 Check
PLAT410_ALERT_2_G Short Intra H...H Contact  H8       ..H34A     .       1.97 Ang.
                                                       x,y,z  =      1_555 Check
PLAT480_ALERT_4_G Long H...A H-Bond Reported H18B     ..CL2      .       2.93 Ang.
PLAT860_ALERT_3_G Number of Least-Squares Restraints ............          12 Note
PLAT909_ALERT_3_G Percentage of I>2sig(I) Data at Theta(Max) Still        60% Note
PLAT933_ALERT_2_G Number of HKL-OMIT Records in Embedded .res File          3 Note
PLAT978_ALERT_2_G Number C-C Bonds with Positive Residual Density.          5 Info
```

0 **ALERT level A** = Most likely a serious problem - resolve or explain
0 **ALERT level B** = A potentially serious problem, consider carefully
10 **ALERT level C** = Check. Ensure it is not caused by an omission or oversight
16 **ALERT level G** = General information/check it is not something unexpected

3 ALERT type 1 CIF construction/syntax error, inconsistent or missing data
12 ALERT type 2 Indicator that the structure model may be wrong or deficient
6 ALERT type 3 Indicator that the structure quality may be low
4 ALERT type 4 Improvement, methodology, query or suggestion
1 ALERT type 5 Informative message, check

It is advisable to attempt to resolve as many as possible of the alerts in all categories. Often the minor alerts point to easily fixed oversights, errors and omissions in your CIF or refinement strategy, so attention to these fine details can be worthwhile. In order to resolve some of the more serious problems it may be necessary to carry out additional measurements or structure refinements. However, the purpose of your study may justify the reported deviations and the more serious of these should normally be commented upon in the discussion or experimental section of a paper or in the "special_details" fields of the CIF. checkCIF was carefully designed to identify outliers and unusual parameters, but every test has its limitations and alerts that are not important in a particular case may appear. Conversely, the absence of alerts does not guarantee there are no aspects of the results needing attention. It is up to the individual to critically assess their own results and, if necessary, seek expert advice.

Publication of your CIF in IUCr journals

A basic structural check has been run on your CIF. These basic checks will be run on all CIFs submitted for publication in IUCr journals (*Acta Crystallographica*, *Journal of Applied Crystallography*, *Journal of Synchrotron Radiation*); however, if you intend to submit to *Acta Crystallographica Section C* or *E* or *IUCrData*, you should make sure that full publication checks are run on the final version of your CIF prior to submission.

Publication of your CIF in other journals

Please refer to the *Notes for Authors* of the relevant journal for any special instructions relating to CIF submission.

PLATON version of 18/05/2022; check.def file version of 17/05/2022

Datablock sac158 - ellipsoid plot

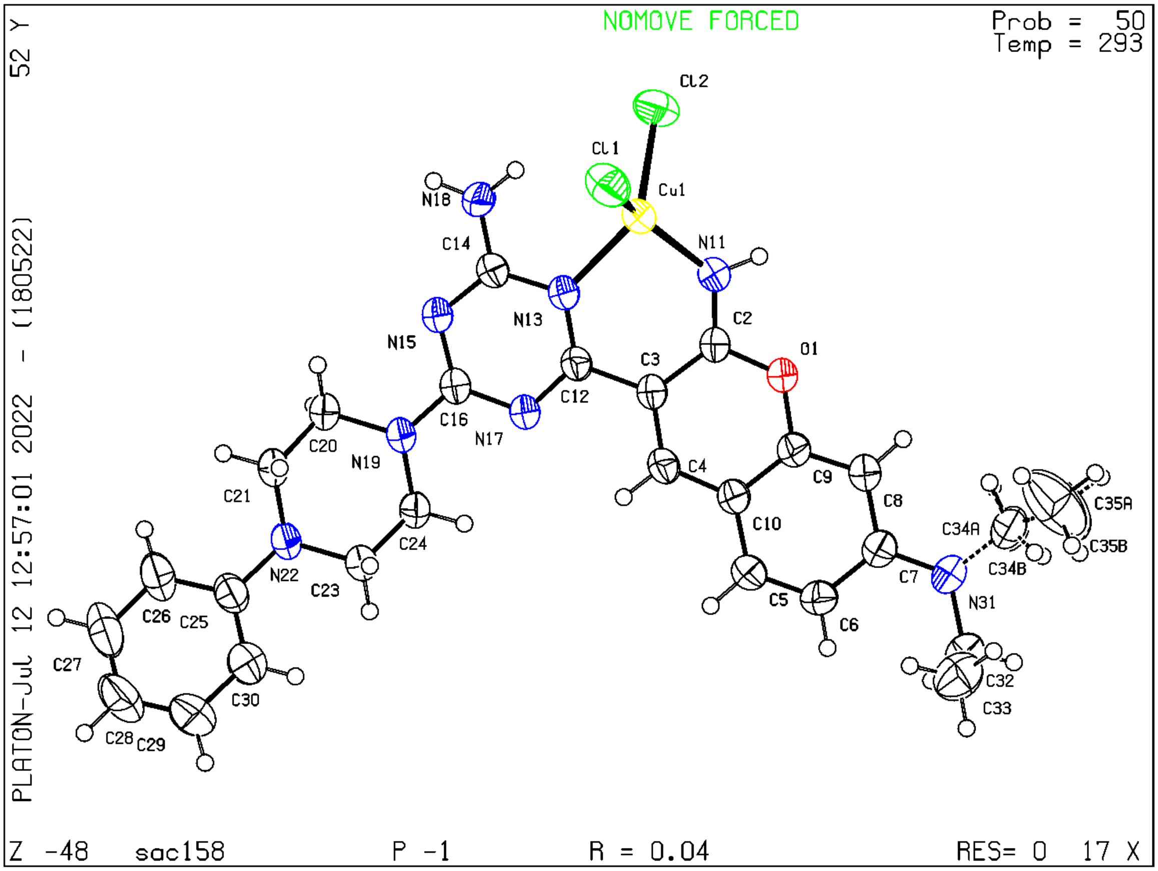

Supplement: Supplementary file 1 [file molecules-27-07155-s001.zip › checkcif-compound_2g_rev.pdf]
