# Supplementary material for: Synthesis, Structure and Cytotoxic Properties of Copper(II) Complexes of 2-Iminocoumarins Bearing a 1,3,5-Triazine or Benzoxazole/Benzothiazole Moiety
Source: Molecules. 2022 Oct 22;27(21):7155. doi: 10.3390/molecules27217155 (PMC9659224; doi:10.3390/molecules27217155)

checkCIF/PLATON report

Structure factors have been supplied for datablock(s) sac156

THIS REPORT IS FOR GUIDANCE ONLY. IF USED AS PART OF A REVIEW PROCEDURE FOR PUBLICATION, IT SHOULD NOT REPLACE THE EXPERTISE OF AN EXPERIENCED CRYSTALLOGRAPHIC REFEREE.

No syntax errors found. CIF dictionary Interpreting this report

Datablock: sac156

```
Bond precision:   C-C = 0.0050 A          Wavelength=1.54184

Cell:            a=16.9813(4)     b=8.8458(2)         c=17.1929(4)
                 alpha=90         beta=109.043(3)     gamma=90
Temperature:     294 K

                Calculated                  Reported
Volume          2441.27(11)                 2441.26(11)
Space group     P 21/n                      P 1 21/n 1
Hall group      -P 2yn                      -P 2yn
Moiety formula  2(C22 H20 Cl3 Cu N7 O), O   C22 H20 Cl3 Cu N7 O, 0.5(O)
Sum formula     C44 H40 Cl6 Cu2 N14 O3      C22 H21 Cl3 Cu N7 O1.50
Mr              1152.70                     577.35
Dx,g cm-3       1.568                       1.571
Z               2                           4
Mu (mm-1)       4.563                       4.563
F000            1172.0                      1176.0
F000'           1171.05
h,k,lmax        20,10,20                    20,10,20
Nref            4320                        4315
Tmin,Tmax       0.849,0.955                 0.624,1.000
Tmin'           0.254

Correction method= # Reported T Limits: Tmin=0.624 Tmax=1.000
AbsCorr = MULTI-SCAN

Data completeness= 0.999           Theta(max)= 66.599

R(reflections)= 0.0453( 3368)                          wR2(reflections)=
                                                       0.1335( 4315)
S = 1.021                    Npar= 328
```

The following ALERTS were generated. Each ALERT has the format
test-name_ALERT_alert-type_alert-level.
Click on the hyperlinks for more details of the test.

Alert level A

```
PLAT430_ALERT_2_A Short Inter D...A Contact  O1W      ..O1W      .       1.82 Ang.
                                            1-x,2-y,-z  =      3_675 Check
```

Author Response: The water molecule with half-occupancy is disordered around inversion center therefore a short intermolecular contact is generated by the program

Alert level B

```
PLAT260_ALERT_2_B Large Average Ueq of Residue Including       O1W      0.442 Check
```

Alert level C

```
PLAT041_ALERT_1_C Calc. and Reported SumFormula    Strings  Differ     Please Check
PLAT042_ALERT_1_C Calc. and Reported MoietyFormula Strings  Differ     Please Check
PLAT043_ALERT_1_C Calculated and Reported Mol. Weight Differ by ..       2.00 Check
PLAT068_ALERT_1_C Reported F000 Differs from Calcd (or Missing)...     Please Check
PLAT410_ALERT_2_C Short Intra H...H Contact  H21A      ..H26      .       1.94 Ang.
                                                      x,y,z  =      1_555 Check
PLAT410_ALERT_2_C Short Intra H...H Contact  H23B      ..H30      .       1.92 Ang.
                                                      x,y,z  =      1_555 Check
PLAT420_ALERT_2_C D-H Bond Without Acceptor  N11      --H11      .     Please Check
PLAT911_ALERT_3_C Missing FCF Refl Between Thmin & STh/L=    0.595          5 Report
```

Alert level G

```
FORMU01_ALERT_1_G  There is a discrepancy between the atom counts in the
            _chemical_formula_sum and _chemical_formula_moiety. This is
            usually due to the moiety formula being in the wrong format.
            Atom count from _chemical_formula_sum:   C22 H21 Cl3 Cu1 N7 O1.5
            Atom count from _chemical_formula_moiety:C22 H20 Cl3 Cu1 N7 O1.5
FORMU01_ALERT_2_G  There is a discrepancy between the atom counts in the
            _chemical_formula_sum and the formula from the _atom_site* data.
            Atom count from _chemical_formula_sum:C22 H21 Cl3 Cu1 N7 O1.5
            Atom count from the _atom_site data:  C22 H20 Cl3 Cu1 N7 O1.5
CELLZ01_ALERT_1_G Difference between formula and atom_site contents detected.
CELLZ01_ALERT_1_G WARNING: H atoms missing from atom site list. Is this intentional?
           From the CIF: _cell_formula_units_Z    4
           From the CIF: _chemical_formula_sum  C22 H21 Cl3 Cu N7 O1.50
           TEST: Compare cell contents of formula and atom_site data

           atom    Z*formula  cif sites diff
           C          88.00     88.00    0.00
           H          84.00     80.00    4.00
           Cl         12.00     12.00    0.00
           Cu          4.00      4.00    0.00
```

```
          N         28.00     28.00    0.00
          O          6.00      6.00    0.00
PLAT002_ALERT_2_G Number of Distance or Angle Restraints on AtSite          5 Note
PLAT012_ALERT_1_G No       _shelx_res_checksum Found in CIF ......     Please Check
PLAT045_ALERT_1_G Calculated and Reported Z Differ by a Factor ...      0.500 Check
PLAT172_ALERT_4_G The CIF-Embedded .res File Contains DFIX Records          1 Report
PLAT232_ALERT_2_G Hirshfeld Test Diff (M-X)  Cu1      --Cl1      .        6.0 s.u.
PLAT300_ALERT_4_G Atom Site Occupancy of O1W        Constrained at        0.5 Check
PLAT302_ALERT_4_G Anion/Solvent/Minor-Residue Disorder (Resd  2  )       100% Note
PLAT304_ALERT_4_G Non-Integer Number of Atoms in ..... (Resd  2  )       0.50 Check
PLAT311_ALERT_2_G Isolated Disordered Oxygen Atom (No H's ?) .....        O1W Check
PLAT860_ALERT_3_G Number of Least-Squares Restraints ............           3 Note
PLAT883_ALERT_1_G No Info/Value for _atom_sites_solution_primary .     Please Do !
PLAT909_ALERT_3_G Percentage of I>2sig(I) Data at Theta(Max) Still        60% Note
PLAT933_ALERT_2_G Number of HKL-OMIT Records in Embedded .res File          2 Note
PLAT978_ALERT_2_G Number C-C Bonds with Positive Residual Density.          2 Info
```

---

 1 **ALERT level A** = Most likely a serious problem - resolve or explain
 1 **ALERT level B** = A potentially serious problem, consider carefully
 8 **ALERT level C** = Check. Ensure it is not caused by an omission or oversight
18 **ALERT level G** = General information/check it is not something unexpected

10 ALERT type 1 CIF construction/syntax error, inconsistent or missing data
11 ALERT type 2 Indicator that the structure model may be wrong or deficient
 3 ALERT type 3 Indicator that the structure quality may be low
 4 ALERT type 4 Improvement, methodology, query or suggestion
 0 ALERT type 5 Informative message, check

It is advisable to attempt to resolve as many as possible of the alerts in all categories. Often the minor alerts point to easily fixed oversights, errors and omissions in your CIF or refinement strategy, so attention to these fine details can be worthwhile. In order to resolve some of the more serious problems it may be necessary to carry out additional measurements or structure refinements. However, the purpose of your study may justify the reported deviations and the more serious of these should normally be commented upon in the discussion or experimental section of a paper or in the "special_details" fields of the CIF. checkCIF was carefully designed to identify outliers and unusual parameters, but every test has its limitations and alerts that are not important in a particular case may appear. Conversely, the absence of alerts does not guarantee there are no aspects of the results needing attention. It is up to the individual to critically assess their own results and, if necessary, seek expert advice.

Publication of your CIF in IUCr journals

A basic structural check has been run on your CIF. These basic checks will be run on all CIFs submitted for publication in IUCr journals (*Acta Crystallographica*, *Journal of Applied Crystallography*, *Journal of Synchrotron Radiation*); however, if you intend to submit to *Acta Crystallographica Section C* or *E* or *IUCrData*, you should make sure that full publication checks are run on the final version of your CIF prior to submission.

Publication of your CIF in other journals

Please refer to the *Notes for Authors* of the relevant journal for any special instructions relating to CIF submission.

PLATON version of 18/05/2022; check.def file version of 17/05/2022

Datablock sac156 - ellipsoid plot

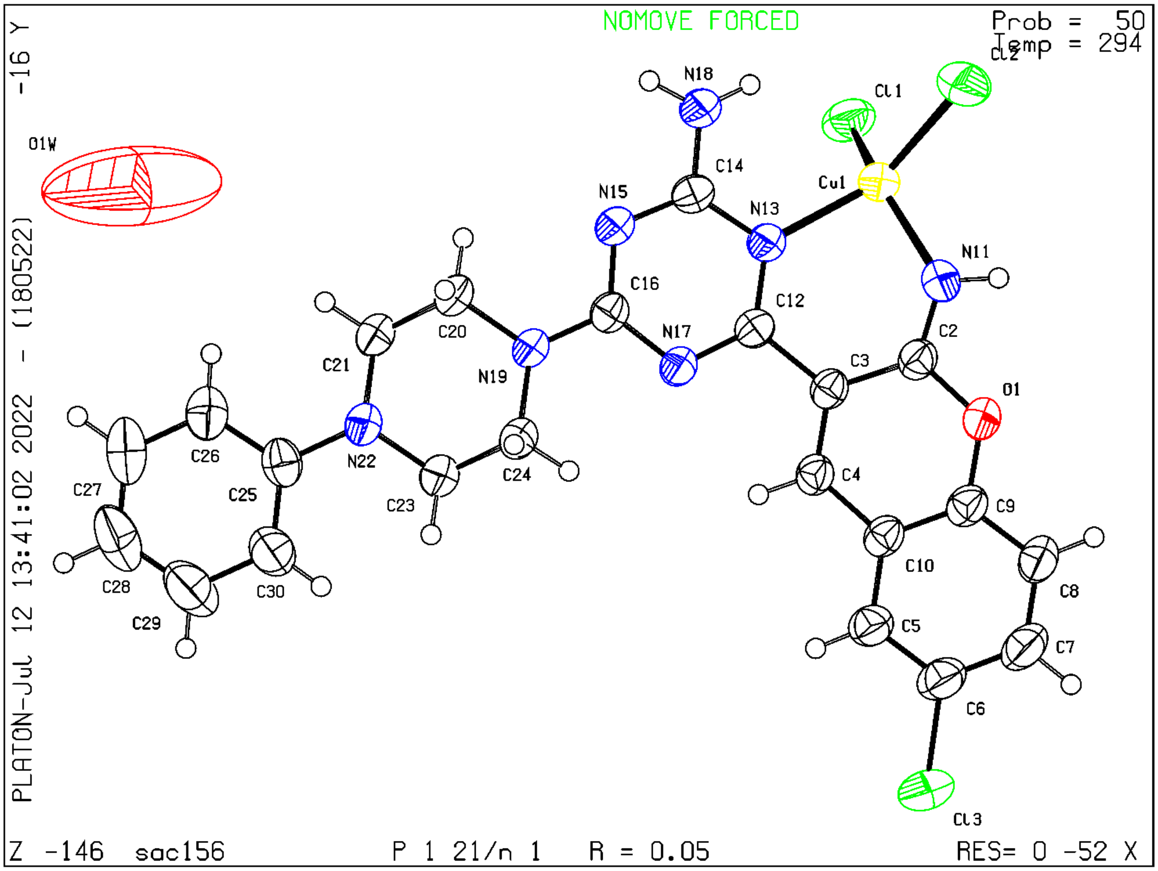

Supplement: Supplementary file 1 [file checkcif-comound_2h_rev.pdf › checkcif-comound_2h_rev.pdf]
